# The Influence of the Comonomer Ratio and Reaction Temperature on the Mechanical, Thermal, and Morphological Properties of Lignin Oil–Sulfur Composites

**DOI:** 10.3390/molecules29174209

**Published:** 2024-09-05

**Authors:** Katelyn A. Tisdale, Nawoda L. Kapuge Dona, Rhett C. Smith

**Affiliations:** Department of Chemistry and Center for Optical Materials Science and Engineering Technology, Clemson University, Clemson, SC 29634, USA

**Keywords:** sustainable composites, sulfur, lignin, lignin oil, compressive strength

## Abstract

Although lignin is a plentiful biomass resource, it continually exists as an underutilized component of biomass material. Elemental sulfur is another abundant yet underutilized commodity produced as a by-product resulting from the refining of fossil fuels. The current study presents a strategy for preparing five durable composites via a simple one-pot synthesis involving the reaction of lignin oil and elemental sulfur. These lignin oil–sulfur composites LOS_x_@T (where *x* = wt. % sulfur, ranging from 80 to 90, and T represents the reaction temperature in °C) were prepared via the reaction of elemental sulfur and lignin oil (LO) with elemental sulfur. The resulting composites could be remelted and reshaped several times without the loss of mechanical strength. Mechanical, thermal, and morphological studies showed that LOS_x_@T possesses properties competitive with some mechanical properties of commercial building materials, exhibiting favorable compressive strengths (22.1–35.9 MPa) and flexural strengths (5.7–6.5 MPa) exceeding the values required for many construction applications of ordinary Portland cement (OPC) and brick formulations. While varying the amount of organic material did not result in a notable difference in mechanical strength, increasing the reaction temperature from 230 to 300 °C resulted in a significant increase in compressive strength. The results reported herein reveal potential applications of both lignin and waste sulfur during the ongoing effort toward developing recyclable and sustainable building materials.

## 1. Introduction

**Figure 1:**
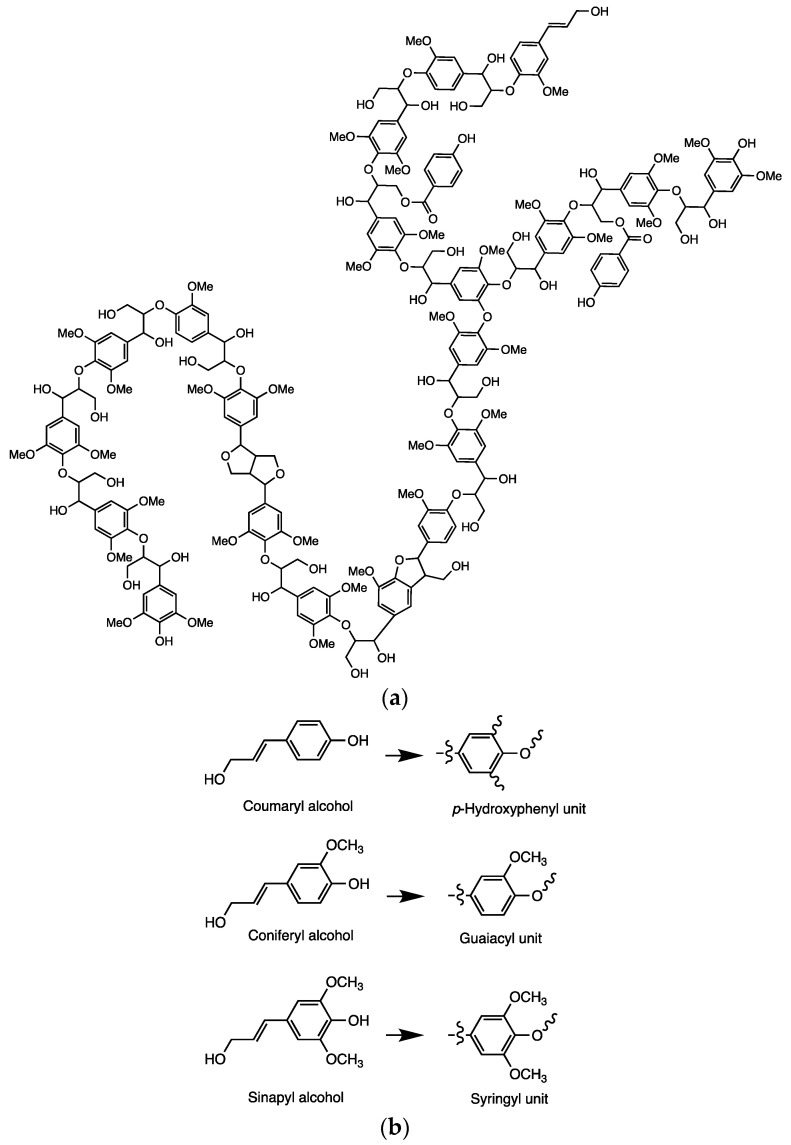
Structure displaying a lignin polymer (**a**) and lignin biosynthetic precursors (**b**).

Lignin holds about 30% of the Earth’s carbon [1,2,3,4] and is a widely available potential feedstock for the chemical industry. Biomass processing produces over 5 billion mt of waste lignin annually, and paper production contributes a supplementary 600,000 tons/year of lignin waste [5]. Despite the high abundance of lignin, its complex structure (Figure 1a) [6,7,8,9,10] introduces many challenges when developing methods for its valorization [11,12,13,14,15,16]. Lignin is composed of mixtures of coniferyl alcohol, 4-*p*-coumaryl alcohol, and sinapyl alcohol units linked together, forming the subunits *p*-hydroxyphenyl, guaiacyl, and syringyl, correspondingly, in lignin (Figure 1b) [17,18,19,20,21,22,23,24,25,26,27,28]. The magnitude of the contribution of each component varies with both the plant species and the part of the plant from which the lignin is derived [1,29,30].

Both “upstream” and “downstream” processes contribute to efficient lignin valorization. Upstream processes involve separating and isolating lignin, lignin bioengineering, and catalytic conversion, whereas downstream methods involve the depolymerization and upgrading of lignin [31,32,33]. Depolymerization yields bio-oils or lignin oils comprising mixtures of small molecular fragments of lignin and is among the most promising routes to lignin employment. Green methods of lignin depolymerization are therefore being hotly pursued. A few “green” solvents employed in extraction processes involve supercritical fluids, liquified gases, and bio-based solvents. Supercritical fluid extraction (SFE) requires an extraction solvent that can be removed easily, a shorter extraction time, and enhanced selectivity and efficiency [34]. Bio-based solvents are derivative of a broad range of biomass sources, including the extraction of vegetable oils, the fermentation of carbohydrates, and the steam distillation of wood [35,36,37]. Liquified gases are another green solvent. They require low temperature for easy evaporation, allowing for room-temperature liquid gas extractions to be performed at room temperature, consuming minimal energy and extracts containing an insignificant amount residual solvents [38,39]. A recent report [40] discussing lignin’s mild thermolytic solvolysis of yielding a solubilized form of lignin (termed lignin oil) garnered particular interest for the present study due to the process being performed at temperatures ranging from 100 to 350 °C by means of various alcohols as solvents [41]. By heating in an ethanol solvent at a low temperature of 100 °C, this procedure accessibly accomplished the conversion of lignin (64 wt. %) to the desired product, lignin oil (LO). Ethanol is a preferred green solvent and is advantageous because of its low cost and availability [42,43,44,45]. Lignin oil produced by this reported procedure was used in the studies described herein.

Another, existing abundant, and underutilized by-product is elemental sulfur. Over 80 Mt/y of elemental sulfur is produced from fossil fuel refining [46,47,48,49,50,51,52,53,54,55,56,57,58,59,60,61,62]. Multiple efforts have been previously reported for preparing composites from lignin or lignin derivatives and elemental sulfur [63,64,65,66,67].

Early examples of high-sulfur-content materials (HSMs) were made using the inverse vulcanization [57,68,69,70,71,72,73,74,75,76,77,78,79,80,81,82] of olefins [64,83,84,85,86,87,88,89,90,91,92]. The preparation of lignin–sulfur composites thus required the modification of lignin with olefin-bearing substituents before the reaction with sulfur could take place [63,65]. The previously reported LS_x_ (where *x* = wt. % sulfur, varying between 80 and 99), for example, was prepared using allyl-derivatized lignin (10 wt. %) [63], while ELS_x_@T (where *x* = wt. % sulfur, varying between 80 and 90, and T represents reaction temperature in °C) was prepared by reactions of oleic-esterified lignin with sulfur [65]. Two reaction temperatures were used for the preparation of ELS_x_@T due to the fact that the reaction at 180 °C strictly produces S–C bonds at olefinic sites, while reacting at a higher temperature of 230 °C can produce S–C bonds at both olefinic sites and aryl sites [65]. Although LS_x_ and ELS_x_@T proved to be durable lignin–sulfur composites, the multistep synthesis required the use of solvents and separation methods that detracted from the desired greenness, affordability, and process atom economy related to these materials.

The preparation of CLS_90_ (90 wt. % S, 10 wt. % chlorolignin, a paper/pulping waste product) [93] via the reaction of sulfur (90 wt. %) with chlorolignin (10 wt. %) through radical-induced aryl halide–sulfur polymerization (RASP) [94] presented a more proficient one-pot synthesis to produce a durable composite [95]. However, this process yields 0.5 equivalents of toxic S_2_Cl_2_ for each C–S bond formed, making it less environmentally friendly and less atom-economical compared to composites prepared via inverse vulcanization. A wide range of composites incorporating lignin have also been reported that do not require sulfur [96,97,98,99,100,101,102,103,104,105].

**Scheme 1:**
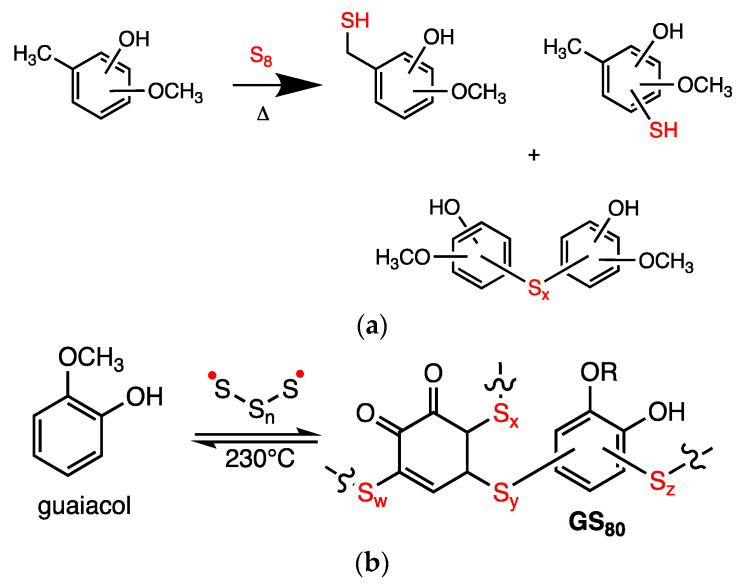
(**a**) S–C bond-forming reactions for lignin-derived small molecules bearing some combination of −CH_3_, −OH, and −OCH_3_ substituents [107] and (**b**) the major S–C bond forming reaction of composite GS_80_ from sulfur and guaiacol. Polymeric sulfur is represented by –S_x_– with subscript letters w, x, y, and z signifying some amount of sulfur in the polymer chain.

The search for a more sustainable S–C bond forming reaction to allow the direct utilization of lignin without the need for olefination has simplified the synthetic process toward lignin–sulfur composites and has improved the atom economy of the processes used for their preparation. Recently reported C–S bond-forming mechanisms during the reaction of sulfur with anisole derivatives (Scheme 1) such as *O*,*O*′-dimethylbisphenol A [106], guaiacol [64], and others [107] suggest that the direct reaction of lignin oil mixtures with sulfur was possible, as was validated in a recent proof-of-principle study detailing the crosslinking that takes place between sulfur and small lignin derivatives present in lignin oil [108].

Herein, five composites were prepared by heating lignin oil (LO) with elemental sulfur for 2 h. The composites, including LOS_x_@T (where *x* = wt. % sulfur, varying from 80 to 90, and T represents the reaction temperature in °C), were of interest to exploit the features of previously reported ELS_90_@180 and ELS_90_@230 due to S–C bond-forming at both olefinic and aryl sites after an increase in temperature. Previously studied model compounds with functional groups found within thermal degradation products or lignin subunits were reacted with elemental sulfur, implementing matching conditions [107] as reported to enhance the understanding of S–C bond-forming reactions within the lignin–sulfur composites prepared herein (Scheme 1a). The thermal, morphological, and mechanical properties were analyzed using powder X-ray diffraction (XRD), scanning electron microscopy accompanied by elemental mapping via energy-dispersive X-ray analysis (SEM-EDX), thermogravimetric analysis (TGA), flexural strength analysis, mechanical test stand analysis, and differential scanning calorimetry (DSC).

## 2. Results and Discussion

### 2.1. Synthesis and Chemical Characterization of Composites

The lignin oil utilized herein was prepared and characterized as previously reported [108].

**Figure 2:**
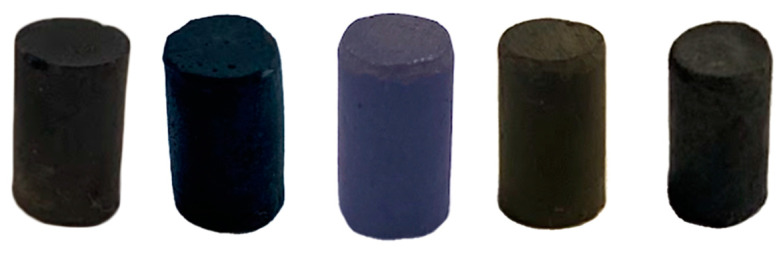
Representative photos of compressional cylinders of (from left to right) LOS_80_@230, LOS_85_@230, LOS_90_@230, LOS_90_@250, and LOS_90_@300.

**Figure 3:**
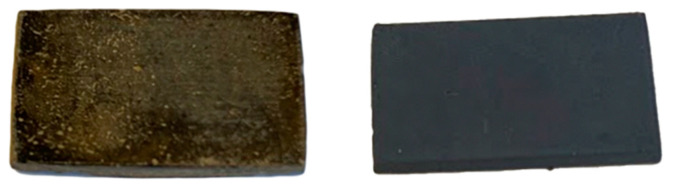
Representative photos of rectangular prisms for flexural strength tests of LOS_90_@230 (**left**) and LOS_85_@230 (**right**).

Composites LOS_x_@T (where *x* = wt. % sulfur, varying from 80 to 90, and T represents the reaction temperature in °C) were prepared by heating lignin oil (10, 15, or 20 wt. %) and sulfur (90, 85, or 80 wt. %) to the requisite temperature in a sealed vessel for 2 h. The composites LOS_80_@230 and LOS_85_@230, and LOS_90_@230 [108] were prepared in thick-walled glass pressure vessels slowly heated to 230 °C and then held at 230 °C for 2 h. Composites LOS_90_@250 and LOS_90_@300 were prepared in a stainless steel autoclave reactor The composites were mechanically stirred and slowly heated to either 250 °C or 300 °C, respectively, and held at that temperature for 2 h. After cooling to room temperature, all the materials solidified into a dark brown, remeltable solid. After melting, the composite material was transferred into molds for shaping and cooled to room temperature (Figure 2 and Figure 3).

The sulfur present in high sulfur-content materials (HSMs) exists as both –S_x_– chains that are linked covalently to the organic comonomers and entrapped sulfur species such as *cyclo*-S_8_, known as “dark sulfur”, which is not linked covalently to the organic comonomers [109,110,111]. Dark sulfur’s relative quantity within an HSM can influence both its thermal and its mechanical properties. In this study, dark sulfur was extracted from each HSM using ethyl acetate. The wt. % of each sample that was soluble (dark sulfur) and the insoluble fraction (organic crosslinked network) are provided in Table 1. The majority of sulfur in the reported lignin–sulfur composites was stabilized as covalently attached crosslinking catenates, with all of the composites showing a similar contribution of dark sulfur over a narrow range of 22–31 wt. %. Elemental analysis confirmed that very little organic material was extractable, with soluble portions consisting of 94–99% sulfur. It is important to note that the extraction experiment was only conducted to assess the relative contributions of the dark sulfur and of the crosslinked network to the overall structure. All subsequent thermal, morphological, and mechanical testing was conducted on the complete composites, comprising the network with the entrapped dark sulfur.

**Figure 4:**
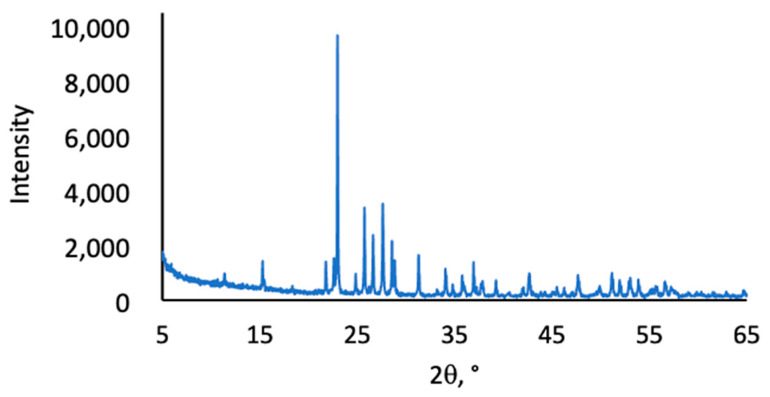
Powder XRD data for LOS_90_@230.

**Figure 5:**
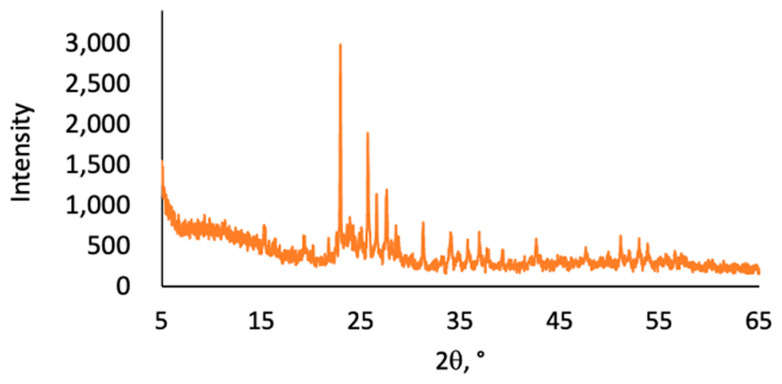
Powder XRD data for LOS_85_@230.

**Figure 6:**
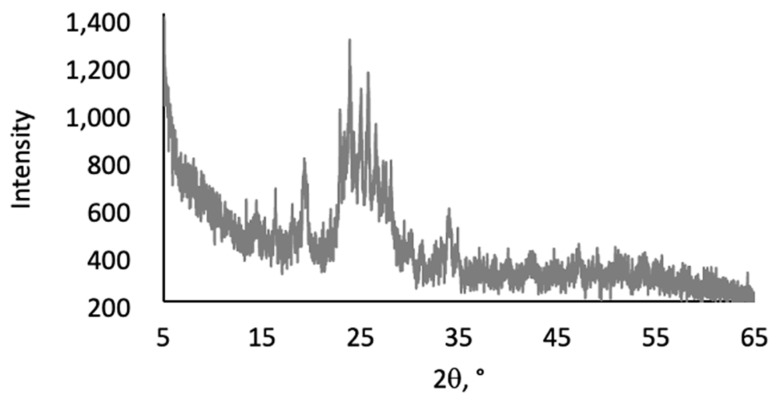
Powder XRD trace for LOS_80_@230.

Powder X-ray diffraction (PXRD) (Figure 4, Figure 5, Figure 6 and Figures S1–S5, Supplementary Materials) was used for the initial qualitative assessment of crystallinity and species contributing to the crystalline domains of LOS_x_@T composites. As the amount of organic material increased, the PXRD data reveal the anticipated corresponding reduction in crystallinity: LOS_90_@230 is a crystalline polymer, LOS_85_@230 is a partially amorphous polymer, and LOS_80_@230 is primarily amorphous. This trend reflects the diminished capacity for the crystalline packing of poly/oligosulfur crosslinking chains as they become progressively shorter in response to the availability of more organic crosslinkable sites. The sharp peaks are the crystalline sulfur, while broad baselines between 20 and 30 2θ, most noticeable for the amorphous LOS_80_@230 (Figure 6), are characteristic of the polymer. LOS_90_@230 is almost entirely crystalline sulfur within the polymer composite, as seen by the very tall, sharp peaks (Figure 4). While PXRD provides qualitative confirmation of a decreasing percentage of crystallinity with increasing organic comonomer, it only provides a semiquantitative estimate of the percentage of crystallinity. Due to the nature of the composite having numerous diffractions of crystalline sulfur, it is difficult to identify lignin’s contribution within the pattern. Therefore, differential scanning calorimetry (DSC) data were used to calculate a more accurate percentage crystallinity for the composites (Table 2). We find that the percentage crystallinity values correlate quantitatively well with the PXRD patterns, which show increasing intensity of broad features about 20–30 2θ.

**Figure 7:**
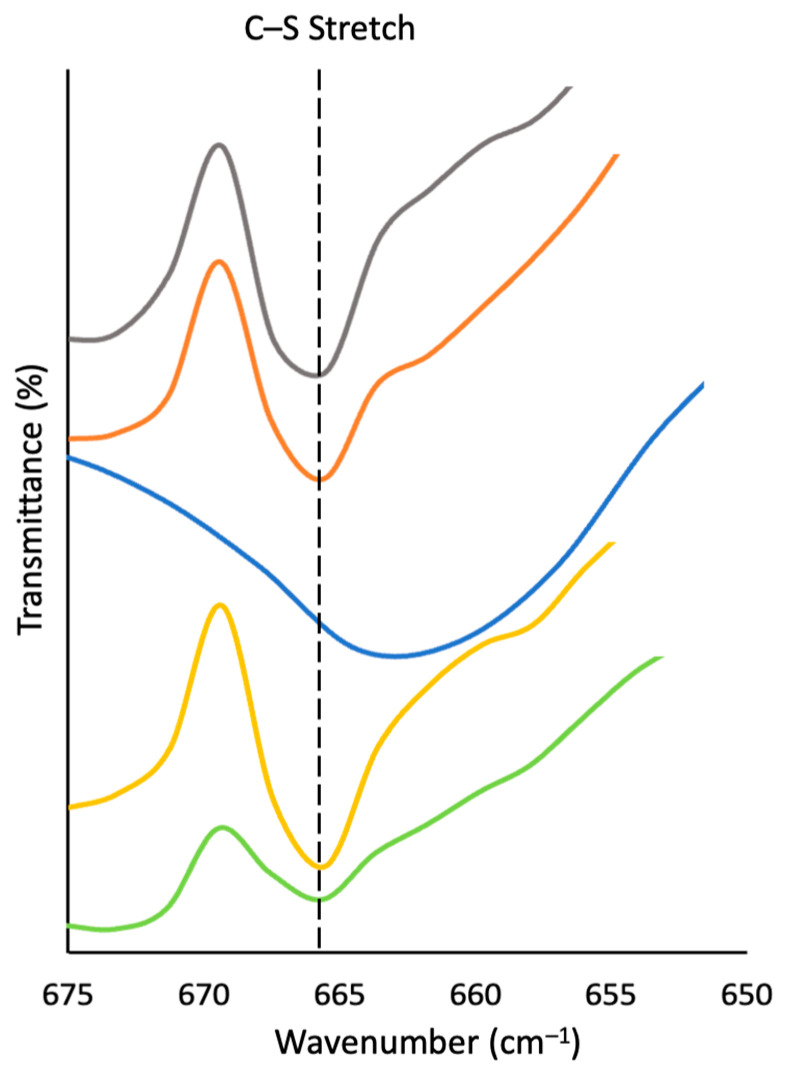
Portion of the FT-IR spectrum revealing the S–C stretch for LOS_80_@230 (grey trace), LOS_85_@230 (orange trace), LOS_90_@230 (blue trace), LOS_90_@250 (yellow trace), and LOS_90_@300 (green trace). The entire set of IR spectra are presented in the Supplementary Materials file.

FT-IR spectra revealed further evidence of the formation of C–S bonds between the polymeric sulfur and lignin oil components (Figure 7). At 660 cm^−1^, a visible peak indicates a C–S stretch [112,113]. The IR spectra (Figures S6 and S7, Supplementary Materials) also revealed that LOS_x_@T composites preserved peaks characteristic of LO such as O–H stretches (3200–3500 cm^−1^), C–H stretches (2930 cm^−1^), and C–O stretches (1030–1200 cm^−1^) [114,115]. Other important peaks to note are those characteristic of lignin bands such as the C=O aromatic skeletal vibration observed from 1600 to 1620 cm^−1^ and the unconjugated carbonyl groups observed from 1700 to 1720 cm^−1^. As previously reported, the C–S stretch for LOS_90_@230 is predictably broadened because it has the lowest organic content and reaction temperature of the composites and therefore the longest average polysulfur chains between organic sites, leading to the greatest polydispersity of the possible bond stretch energies. The displacement of the bands observed can also be attributed to the reaction temperatures of the different composites. For example, slightly sharper peaks were observed in LOS_80_@230, LOS_85_@230, and LOS_90_@230 from 600 to 1200 cm^−1^ (Figure S8, Supplementary Materials) when compared to LOS_90_@250 and LOS_90_@300, which were reacted at higher temperatures.

**Figure 8:**
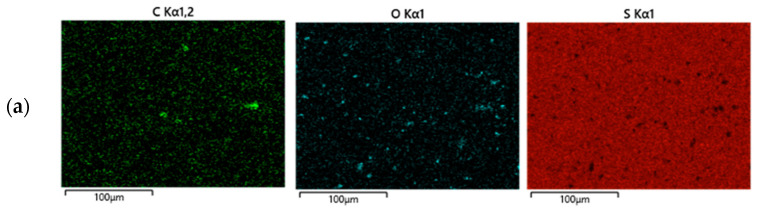

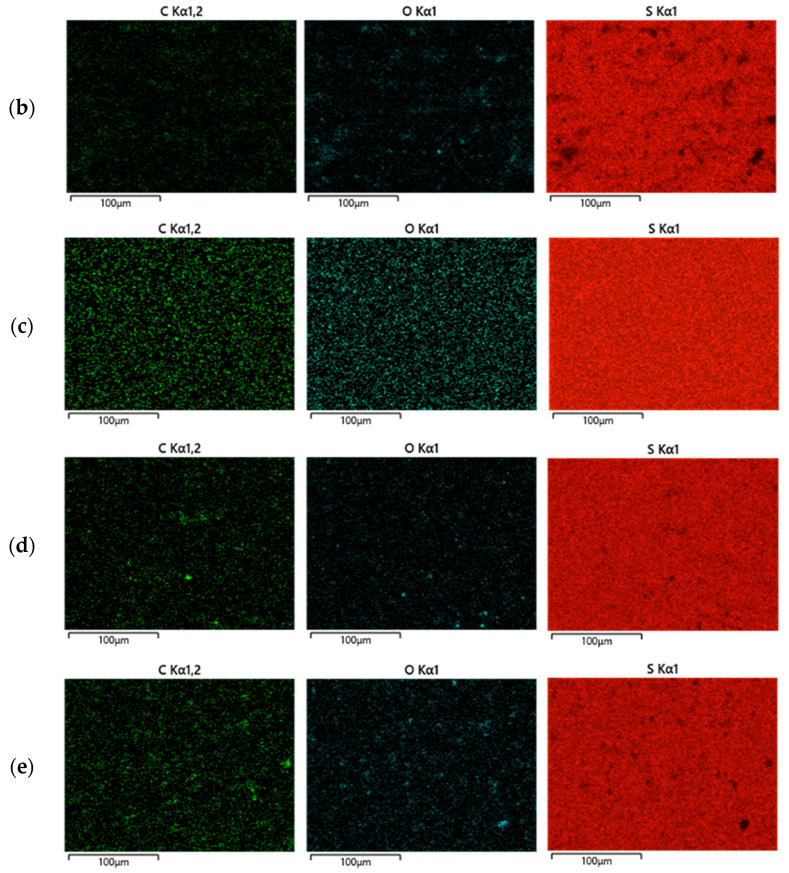
Elemental mapping of the elements carbon (green, left), oxygen (blue, middle), and sulfur (red, right) in (**a**) LOS_80_@230, (**b**) LOS_85_@230, (**c**) LOS_90_@230, (**d**) LOS_90_@250, and (**e**) LOS_90_@300 by energy-dispersive X-ray analysis (EDX).

Scanning electron microscopy (SEM, Figures S8–S12) imaging accompanied by element mapping by energy-dispersive X-ray analysis (EDX) was utilized to evaluate the dispersal of elements in the LOS_x_@T composites (Figure 8). SEM/EDX data revealed a homogeneous distribution of sulfur, carbon, and oxygen.

### 2.2. Thermal and Morphological Properties of LOS_x_@T

**Figure 9:**
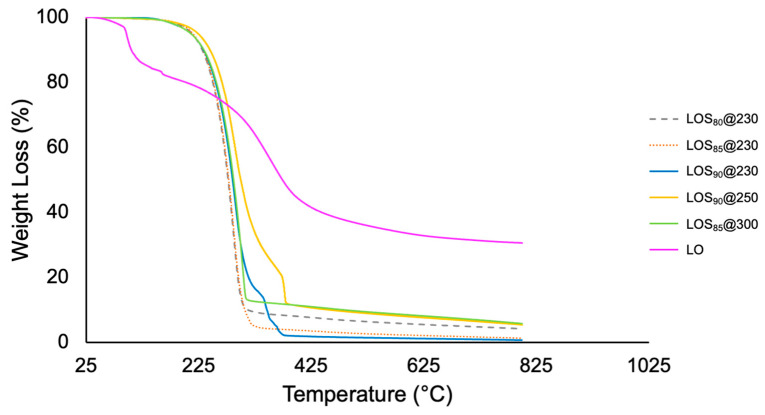
Thermogravimetric analysis (TGA) traces for LOS_80_@230 (grey dashed line), LOS_85_@230 (orange dotted line), LOS_90_@230(blue solid line), LOS_90_@250 (yellow solid line), LOS_90_@300 (green solid line), and lignin oil (LO) (pink solid line).

The thermal stability of the reported LOS_x_@T composites was assessed using thermogravimetric analysis (Figure 9). The TGA data for lignin oil (LO) prior to its reaction with elemental sulfur are also provided in Figure 9, and the origin of the decomposition events in LO have been previously delineated [116]. The TGA trace for each of the composites showed a single decomposition temperature (*T*_d_) ranging from 229 to 231 °C. These temperatures are characteristic of such HSMs due to the fact that elemental sulfur has a *T*_d_ of 229 °C, which is attributable to the sublimation of sulfur in the LOS_x_@T composites. The smaller decomposition event at >300 °C in LOS_90_@230 and LOS_90_@250 is likely attributable to similar mechanisms leading to the corresponding decomposition events observed in lignin oil. The *T*_d_ values for LOS_x_@T and other previously reported lignin-containing HSMs are summarized in Table 2.

**Figure 10:**
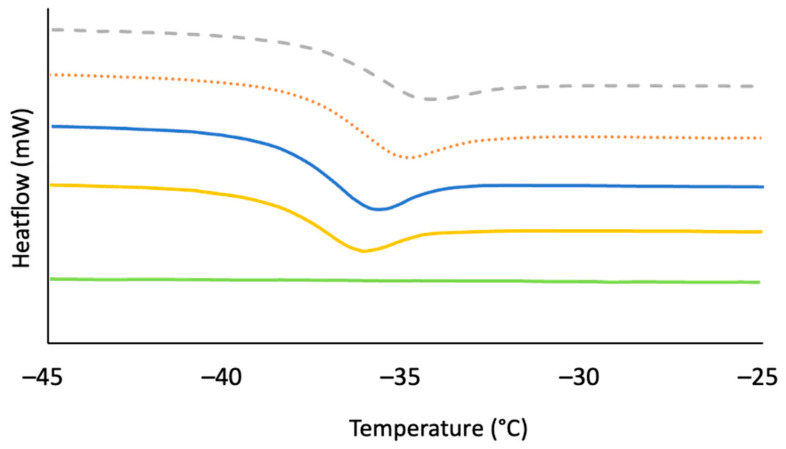
Inset of differential scanning calorimetry (DSC) thermograms emphasizing the glass transition temperature (*T*_g_) of LOS_90_@230 (blue solid line), LOS_85_@230 (orange dotted line), LOS_80_@230 (grey dashed line), LOS_90_@250 (yellow solid line), and LOS_90_@300 (green solid line). Full DSC thermographs from −60 to 140 C° are provided in the Supplementary Information file.

The analysis of LOS_x_@T composites by differential scanning calorimetry (DSC) (Figures S13–S27, Supplementary Materials) showed thermal transitions that were concomitant with the presence of polymeric sulfur and *cyclo*-S_8_. LOS_90_@230, LOS_90_@250, and LOS_90_@300 exhibited the melting peaks (114 °C, 117 °C, and 119 °C) expected for *cyclo*-S_8_. However, LOS_85_@230 and LOS_80_@230 exhibited lower melting peaks at 106 °C and 105 °C, indicating an α-to-β transition of sulfur. A glass transition temperature (*T*_g_) was also witnessed (Figure 10) at −35 °C or −36 °C for all composites other than LOS_90_@300, diagnostic for polymeric sulfur [117,118,119]. Similar to the previously studied LS_90–99_ composites, LOS_90_@300 did not exhibit a glass transition temperature. This is likely due to the higher temperature allowing for more crosslinking, leading to shorter sulfur chains.

Melting and cold crystallization enthalpies determined from DSC data were employed for the calculation of the percentage crystallinity of the composites, further indicating the presence of *cyclo*-S_8_, using Equation (1).

(1) $$\Delta \chi_{c}=1-\left\{\frac{\Delta H_{m}-\Delta H_{cc\;}}{\Delta H_{m\left(S\right)}-\Delta H_{cc\left(S\right)}}\right\}\times 100\%\;$$

where Δ*X_c_* signifies the change in the percentage crystallinity with respect to sulfur, Δ*H_m_* signifies the melting enthalpy of the composite, Δ*H_cc_* represents the cold crystallization enthalpy of the composite, Δ*H_m_*_(*S*)_ is sulfur’s melting enthalpy, and Δ*H_cc_*_(*S*)_ is sulfur’s cold crystallization enthalpy.

**Figure 11:**
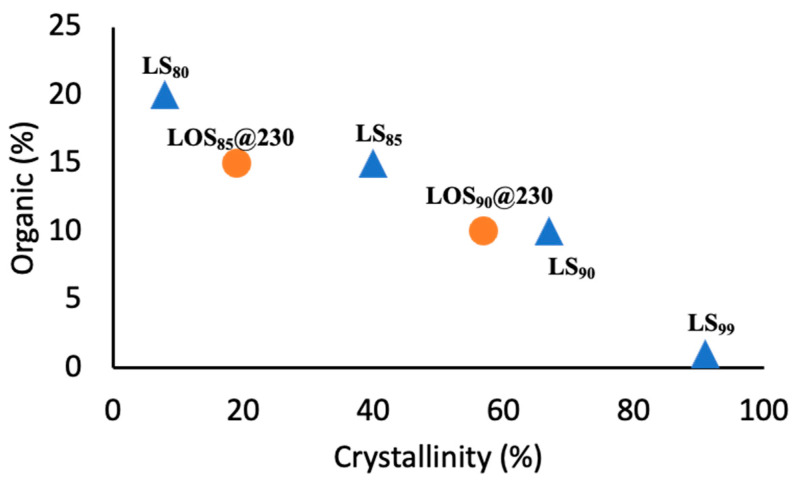
A graphical representation of the general trend of the effect increasing the percentage of organic material has on the percentage crystallinity of LS_x_ (blue triangles) and LOS_x_@T (orange circles).

The percentage crystallinity of LOS_90_@230 is 57% relative to the crystallinity of pure elemental sulfur. Lower percentage crystallinity results in the reduced brittleness of HSMs. Compared to LS_x_ composites (Table 1), LOS_90_@230 possesses a lower percentage crystallinity than any of the composites made from allylated lignin, for example, LS_90–99_ (percentage crystallinity of 67–91%). However, LOS_90_@230 possesses a higher percentage crystallinity than that of composites LS_80_ (8%) and LS_85_ (40%), reflecting the trend more qualitatively revealed by PXRD data (vide supra). These trends are summarized in Figure 11. LOS_85_@230 also has a lower percentage crystallinity (19%) than that of LOS_90_@230 (57%) and LS_85–99_ (40–91%). However, LOS_85_@230 possesses a higher percentage crystallinity than that of composite LS_80_ (8%). The reaction temperature does not seem to have a significant effect on the percentage crystallinity of lignin–sulfur materials.

### 2.3. Mechanical Properties and Environmental Impact Considerations

**Figure 12:**
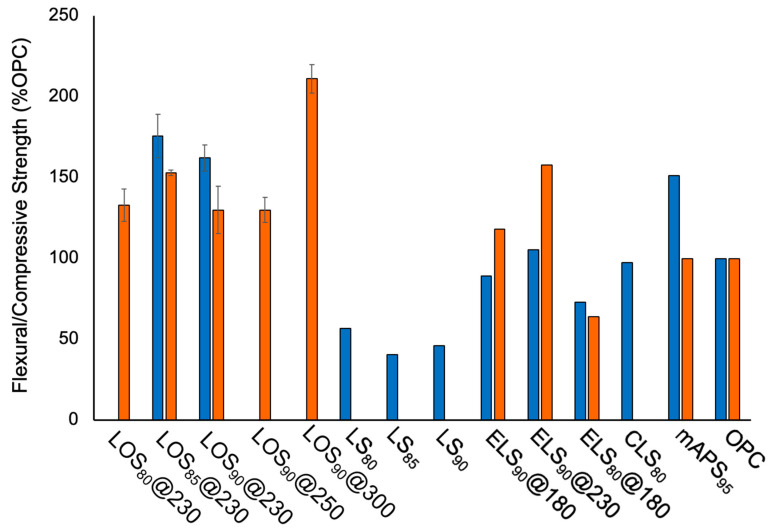
Graphical representation comparing the flexural strength (blue bars) and compressive strength (orange bars) of LOS_x_@T composites with lignin–sulfur composites (LS_x_), lignin/cellulose-containing sulfur composites (ELS_x_@T, CLS_80_, mAPS_95_), and ordinary Portland cement (OPC). Bars not displayed signify that the attendant value was not previously reported for the listed material or could not be obtained for the current research.

**Figure 13:**
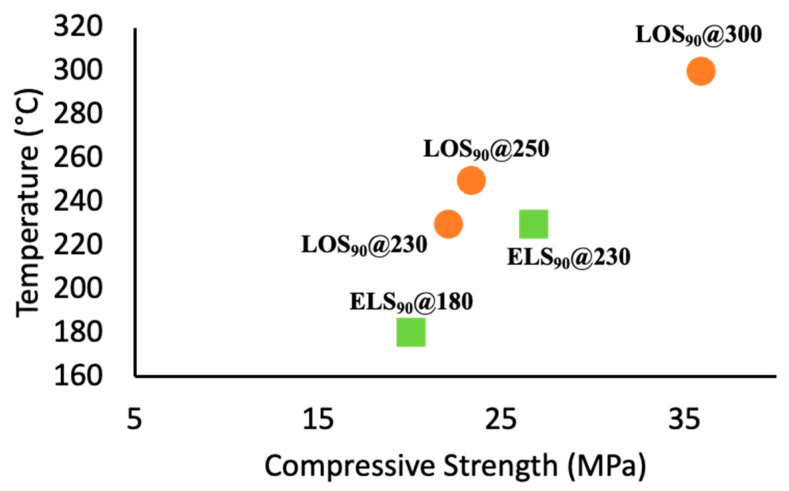
A graphical representation of the general trend of the effect increasing reaction temperature has on the compressive strength of ELS_x_@T (green squares) and LOS_x_@T (orange circles).

The LOS_x_@T composites studied herein must meet the specified mechanical strength requirements to be considered as potential replacements for less sustainable materials. For example, OPC requires a compressive strength greater than 17 MPa and a flexural strength greater than 3 MPa. The compressive strength of the LOS_x_@T composites reported exceeded that required of OPC with strengths ranging from 22.1 to 35.9 MPa. Other lignin-containing HSMs have been reported and compared to the LOS_x_@T composites. The composites ELS_x_@T (where *x* = wt. % sulfur in the reaction mixture and T represents the reaction temperature in °C), for example, were prepared by reacting sulfur with oleic-esterified lignin. ELS_80_@180 has a notably lower compressive strength (10.9 MPa) than LOS_x_@T composites. ELS_80_@180 comprises twice the amount of organic component as LOS_90_, LOS_90_@250, and LOS_90_@300. ELS_80_@180 was also reacted at a much lower temperature in comparison to LOS_x_@T composites, whose reaction temperatures ranged from 230 to 300 °C. A larger amount of organic material and lower reaction temperature possibly contributes to a lower sulfur rank, resulting in a lower compressive strength. The ELS_90_@180 and ELS_90_@230 composites exhibited similar compressive strengths as the LOS_90_@230, LOS_85_@230, LOS_80_@230, and LOS_90_@250 composites. The LOS_90_@300 exhibited a much higher compressive strength (35.9 MPa) than all other composites reported in Table 3 and compared graphically in Figure 12 (stress–strain plots are presented in Figures S28–S32, Supplementary Materials). This can possibly be attributed to the higher reaction temperature of 300 °C, which can lead to a higher sulfur rank and a higher compressive strength [65]. While there was not a clear trend displaying how an increase in organic material affects the compressive strength, an increase in compressive strength is observed as the reaction temperature is increased (Figure 13). The preparation of the oleic-esterified lignin (the precursor for ELS_x_@T) is a much more time- and energy-intensive process compared to the process for preparing the lignin oil required for preparing LOS_x_@T composites. Therefore, preparing lignin-oil-containing HSMs is a more realistic concept for acquiring comparable compressive strength features.

The flexural strengths/moduli of LOS_90_@230 and LOS_85_@230 composites were tested and assessed to reveal high flexural strengths (5.7 MPa and 6.5 MPa) and moduli (186 and 236 MPa) at room temperature (Table 3, stress–strain curves are provided in Figures S33 and S34, Supplementary Materials). These flexural strengths and moduli are much higher than the flexural strengths of previously reported LS_x_ (where *x* = wt. % sulfur, prepared by the inverse vulcanization of allylated lignin) composites with flexural strengths ranging from 1.5 to 2.1 MPa and moduli ranging from 57 to 87 MPa. LOS_90_@230 and LOS_85_@230 also exhibited higher flexural strengths than ELS_x_@T composites (2.7–3.3 MPa), as well as CLS_80_ (3.6 MPa), a chlorolignin–sulfur composite comprising 80 wt. % sulfur. However, LOS_90_@230 exhibited a similar flexural strength to that of mAPS_95_ (5.6 MPa), a composite prepared by the inverse vulcanization of allyl lignin (2 wt. %), allyl cellulose (3 wt. %), and 95 wt. % sulfur. The flexural strengths/moduli were previously reported for additional HSM polymers containing organic crosslinkers. For example, dicyclopentadiene (DCPD), an industrial, inexpensive by-product, forms S-DCPD (50 wt. % dicyclopentadiene and 50 wt. % sulfur) after reaction with elemental sulfur. LOS_85_@230 (6.5 MPa) and LOS_90_@230 (5.7 MPa) compare moderately to S-DCPD (6.0 MPa). However, S-DCPD experiences a decrease in flexural strength when organic crosslinkers are incorporated. Linseed oil reacts with DCPD and sulfur to yield S-DCPD–linseed (50 wt. % sulfur, 25 wt. % dicyclopentadiene, and 25 wt. % linseed oil) and exhibits a faintly lower flexural strength (4.7 MPa). However, when a cheap biomass material known as limonene reacts with DCPD and sulfur to yield S-DCPD–limonene (25 wt. % dicyclopentadiene, 25 wt. % limonene, and 50 wt. % sulfur), a considerably lower flexural strength is revealed (1.9 MPa). Conversely, the flexural moduli of S-DCPD–linseed (1250 MPa), S-DCPD (3700 MPa), and S-DCPD–limonene (1750 MPa) [120] considerably surpass those of LOS_85_@230 (236 MPa) and LOS_90_@230 (186 MPa). Another important observation to note is that the flexural strengths of both the LOS_90_@230 and LOS_85_@230 composites exceed OPC’s flexural strength (3.7 MPa), therefore giving competition for commercial building materials. The mechanical strength characteristics of the LOS_x_@T and previously reported lignin-containing HSMs are summarized in Table 3 and compared graphically in Figure 12.

## 3. Materials and Methods

### 3.1. Materials

Sulfur powder (99.5%) was bought from Alfa Aesar (Haverhill, MA, USA). The chemicals employed did not undergo any further purification. The lignin oil used herein was produced via a reported method implementing the thermal solvolysis of kraft lignin (Sigma Aldrich, St. Louis, MO, USA) in the solvent ethanol following the reported procedure [40]. A detailed description of the preparation and characterization of lignin oil was previously reported for the synthesis and analysis of LOS_90_@230 [108]. The same lignin oil was used to prepare the remaining LOS_x_@T composites reported herein.

### 3.2. General Considerations and Instrumentation

Fourier transform infrared spectra were acquired using an IR instrument (Shimadzu IRAffinity-1S, Shimadzu Corporation, Columbia, MD, USA) equipped with an ATR attachment. Scans were collected over the range of 400–4000 cm^−1^ at ambient temperature with a resolution of 8 cm^−1^.

SEM was attained using a Schottky Field Emission Scanning Electron Microscope SU5000 (Hitachi High-Tech, Tokyo, Japan) operating in variable pressure mode while using an accelerating voltage of 15 kV.

Compressional strength measurements were commenced with cylinders by means of a Mark-10 ES30 (Mark-10 Corporation, Copiague, NY, USA) Manual Test Stand equipped with a Mark 10 M3-200 Force Gauge (USA). The composites studied herein were melted and formed in molds (Smooth-On Oomoo^®^ 25 tin-cure, Oomoo Corp., Richmond, BC, Canada) and then allowed to cool to room temperature. The cylinders stood at room temperature for 4 d before compressive strength was tested. Tests for each composite were performed in triplicates, and the strength reported is the average of the three runs. The long-term stability of the reported materials is unknown.

Flexural strength analyses were performed using a Mettler Toledo DMA 1 STARe System (Mettler Toledo, Columbus, OH, USA) in single-cantilever mode. The samples were formed in silicon resin molds (Smooth-On Oomoo^®^ 25 tin-cure). The sample dimensions were 1.5 × 10.7 × 5.0 mm. The temperature was 25 °C with a clamping force of 1 cN m. Samples were assessed in triplicates, and the observed flexural strengths/moduli are an average of the three trials.

TGA data were recorded (Mettler Toledo TGA 2 STARe System, TA Instruments, New Castle, DE, USA) across the temperature range of 20–800 °C at a heating rate of 10 °C·min^−1^ under a flow of N_2_ (100 mL·min^−1^).

DSC data were obtained by means of a Mettler Toledo DSC 3 STARe System (Mettler Toledo, Columbus, OH, USA) across a temperature range of −60 °C to 140 °C while implementing a heating rate of 10 °C·min^−1^ under a flow of N_2_ (200 mL·min^−1^). DSC measurements were conducted over three cooling and heating cycles. Data from the third heating cycle are reported herein, with the first cycle removing solvent impurities. During the third cycle, the glass-transition temperature was observed. DSC data were utilized to calculate the percentage crystallinity using Equation (1).

UV-vis data were collected on Agilent Technologies Cary 60 UV-vis (Agilent Technologies, Inc., Santa Clara, CA, USA) using Simple Reads software (Cary WinUV Scan Application Version 5.1.0.1016) over the range of 400–600 nm. Dark sulfur was quantified by using the extinction coefficient of sulfur at 275 nm along with the absorbance of ethyl acetate soluble fraction of each composite.

### 3.3. Synthesis of LOS_x_@T Composites

LOS_80_@230 and LOS_85_@230 were prepared by adding elemental sulfur (8.005 g, 8.500 g) and lignin oil (2.001 g, 1.503 g) to a heavy-walled pressure flask sealed by a Viton O-ring and a PTFE stopper along with a PTFE stir bar and then placed in an oil bath at 180 °C under continuous magnetic stirring. Next, the temperature was increased to 230 °C. The mixture was heated under rapid continuous stirring for a duration of 2 h. During the reaction time, the reaction mixture appeared homogeneous and turned dark brown in color. Stirring was stopped, and the reaction was removed from the heat. The material was cooled to room temperature, forming a solid dark brown substance. Forming the compressive strength cylinders (Figure 2) and flexural strength rectangular prisms (Figure 3) required remelting the product at 180 °C and then pouring the product into molds, followed by cooling to room temperature. This method resulted in a 99.7% yield for LOS_80_@230 and a 99.8% yield for LOS_85_@230. For LOS_80_@230 elemental analysis, the theoretical values were C 12.86, H 1.22, and S 80; the actual values found were C 10.94, H 0.46, and S 84.85. For LOS_85_@230 elemental analysis, the theoretical were C 9.64, H 0.91, and S 85; the actual values found were C 3.76, H 0.05, and S 94.49. LOS_90_@230 was prepared as previously reported [108].

LOS90@250 and LOS90@300 were prepared by adding elemental sulfur (13.500 g, 13.505 g) and lignin oil (1.501 g, 1.500 g) to a stainless steel autoclave reactor and heated to 250 °C under rapid mechanical stirring. After 2 h, the heat and stirring was stopped, and the autoclave vessel cooled to room temperature. After cooling, the homogeneous, dark brown solid was removed from the reaction vessel. Forming the compressive strength cylinders (Figure 2) required remelting the product at 180 °C and then pouring the product into molds, followed by cooling to room temperature. For LOS_90_@250 elemental analysis, the theoretical values were C 6.43, H 0.61, and S 90; the actual values found were C 7.33, H 0.14, and S 91.24. For LOS_90_@300 elemental analysis, the theoretical values were C 6.43, H 0.61, and S 90; the actual values found were C 5.43, H 0.05, and S 93.06.

### 3.4. General Method for Dark Sulfur Quantification

The method used herein to determine the quantity of dark sulfur is based on a method previously reported by Hasell’s group [109,110]. This method uses absorbance to quantify dark sulfur in HSMs. A small fraction of the composite (6–7 mg) was weighed to +/−0.0001 g precision using a microbalance and placed in a 250 mL volumetric flask with ethyl acetate and stirred for 30 min. This duration of agitation was chosen due to the solubility of elemental sulfur. Ethyl acetate has a lower solubility for sulfur and not oligomeric fractions; therefore, it will extracts dark sulfur within the composite [110]. The solution was then measured using a Carry-UV. A scan was taken over the range of 400–600 nm, and the absorbance at 275 nm was used in the calibration curve that was used to determine the concentration.

### 3.5. General Method for Ethyl Acetate Extractions for Dark Sulfur Quantification

A small amount of each composite (~50 mg) was placed in a vial along with 10 mL of ethyl acetate. This was then stirred for a duration of 1 h at room temperature. After stirring was finished, the vial was left standing still to allow the material to settle, and the ethyl acetate was then pipetted into a different vial. The ethyl acetate was then allowed to evaporate, with the soluble portion remaining in the vial.

## 4. Conclusions

Herein, we have demonstrated that lignin oil containing a mixture of oligomers can readily react with sulfur diradicals at varying amounts (80–90 wt. % S) and relatively low temperatures (230–300 °C). The resulting material formed durable, remeltable composites exhibiting compressive strengths (22.1–35.9 MPa) and flexural strengths (5.7–6.5 MPa) exceeding that of OPC (17 MPa). Increasing the amount of organic material did not result in a drastic change in properties; however, it was shown that increasing the reaction temperature improved the mechanical strength of the composites and decreased the crystallinity, resulting in a less brittle substance. These results show the great potential lignin oil has in the efforts to valorize the abundant yet underutilized lignin biomass.

## Figures and Tables

**Table 1:** Soluble (dark sulfur) and insoluble (crosslinked material) wt. % fractions of lignin–sulfur composites.

Materials	Dark Sulfur ^[a]^ (wt. %)	Crosslinked Material ^[a]^ (wt. %)
LOS_80_@230	22	82
LOS_85_@230	31	77
LOS_90_@230	21	82
LOS_90_@250	24	78
LOS_90_@300	22	81

^[a]^ Sum of soluble and insoluble fractions is greater than 100% due to rounding and errors associated with the measurements.

**Table 2:** Thermal and morphological properties of lignin oil–sulfur composites, elemental sulfur, and other composites.

Materials	$T_{d}^{\left[a\right]}$/°C	$T_{m}^{\left[b\right]}$/°C	$T_{g,DSC}^{\left[c\right]}$/°C	∆*H_m_* J/g	∆*H_cc_* J/g	Percentage Crystallinity ^[d]^
LOS_90_@230 ^[e]^	229	114	−36	26	ND ^[j]^	57
LOS_85_@230 ^[e]^	230	106	−35	35	26	19
LOS_80_@230 ^[e]^	230	105	−35	32	32	1
LOS90@250 ^[e]^	231	117	−36	30	16	30
LOS90@300 ^[e]^	230	119	ND ^[j]^	ND ^[j]^	ND ^[j]^	ND ^[j]^
LS_80_^[f]^	236	118	−34	30	25	8
LS_85_^[f]^	235	118	−35	34	13	40
LS_90_^[f]^	237	117	ND ^[j]^	34	ND ^[j]^	67
LS_95_^[f]^	235	107	ND ^[j]^	37	ND ^[j]^	63
LS_99_^[f]^	233	113	ND ^[j]^	47	ND ^[j]^	91
GS_80_^[g]^	264	ND ^[j]^	−30	ND ^[j]^	ND ^[j]^	ND ^[j]^
ELS_90_@180 ^[h]^	230	117	−37	ND ^[j]^	ND ^[j]^	ND ^[j]^
ELS_90_@230 ^[h]^	234	117	−37	ND ^[j]^	ND ^[j]^	ND ^[j]^
ELS_80_@180 ^[h]^	231	117	−37	ND ^[j]^	ND ^[j]^	ND ^[j]^
S_8_^[i]^	228/229	119	ND ^[j]^	45	ND ^[j]^	100

^[a]^ The temperature at which a mass loss of 5% is observed. ^[b]^ The peak temperature at the peak maximum of the endothermic melting. ^[c]^ Glass transition temperature. ^[d]^ Percentage crystallinity reduction for each composite sample calculated with respect to sulfur (normalized to 100%). ^[e]^ Lignin oil and elemental sulfur (where *x* = wt. % sulfur, varying from 80 and 90, and T represents the reaction temperature in °C). ^[f]^ LS_x_ composites comprising 10 wt. % allyl-derivatized lignin and elemental sulfur, where *x* = wt. % sulfur in monomer feed, ranging from 80 to 99. ^[g]^ Composites consisting of guaiacol (20 wt. %) and elemental sulfur (80 wt. %). ^[h]^ ELS_x_@T composites comprising esterified lignin and elemental sulfur (where *x* = wt. % sulfur in the reaction mixture, varying from 80 to 90, and T represents the reaction temperature in °C). ^[i]^ Elemental sulfur. ^[j]^ Not determined.

**Table 3:** Mechanical properties of the lignin–sulfur composites LOS_x_@T with other previously reported lignin-containing HSMs composites for comparison.

Materials	Compressive Strength (MPa)	Flexural Strength/Modulus (MPa)	Compressive Strength (% of OPC)
LOS_90_@230 ^[a]^	22.1 ± 2.5	5.7/186	130
LOS_85_@230 ^[a]^	26.0 ± 0.3	6.5/236	153
LOS_80_@230 ^[a]^	22.6 ± 1.7	ND ^[k]^	133
LOS90@250 ^[a]^	22.1 ± 1.3	ND ^[k]^	130
LOS90@300 ^[a]^	35.9 ± 1.5	ND ^[k]^	211
LS_80_^[b]^	ND ^[k]^	2.1/87	ND ^[k]^
LS_85_^[b]^	ND ^[k]^	1.5/76	ND
LS_90_^[b]^	ND ^[k]^	1.7/57	ND
LS_95_^[b]^	ND ^[k]^	ND ^[k]^	ND
LS_99_^[b]^	ND ^[k]^	ND ^[k]^	ND
GS_80_^[c]^	ND ^[k]^	ND ^[k]^	ND
ELS_90_@180 ^[d]^	20.1 ± 2.3	3.3/ND ^[k]^	118
ELS_90_@230 ^[d]^	26.8 ± 0.5	3.9/ND ^[k]^	158
ELS_80_@180 ^[d]^	10.9 ± 0.85	2.7/ND ^[k]^	64
CLS_80_^[e]^	ND ^[k]^	3.6/ND ^[k]^	ND ^[k]^
mAPS_95_^[f]^	17.0	5.6/ND ^[k]^	100
OPC ^[g]^	17.0	3.7/580	100
S-DCPD (1:1) ^[h]^	ND	6.0/3700	ND
S-DCPD–linseed oil (2:1:1) ^[i]^	ND	4.7/1250	ND
S-DCPD–limonene (2:1:1) ^[j]^	ND	1.9/1750	ND

^[a]^ LOS_x_@T composites made from lignin oil and elemental sulfur (where *x* = wt. % sulfur, varying from 80 to 90, and T represents the reaction temperature in °C). ^[b]^ LS_x_ composites comprising 10 wt. % allyl-derivatized lignin and elemental sulfur, where *x* = wt. % sulfur in monomer feed, varying from 80 to 99. ^[c]^ Composites comprising guaiacol (20 wt. %) and elemental sulfur (80 wt. %). ^[d]^ ELS_x_@T composites comprising esterified lignin and elemental sulfur (where *x* = wt. % sulfur in the reaction mixture, varying from 80 to 90 and T represents the reaction temperature in °C). ^[e]^ Composites comprising chlorolignin (20 wt. %) and elemental sulfur (80 wt. %). ^[f]^ Composites comprising allyl lignin (2 wt. %), allyl cellulose (3 wt. %) and elemental sulfur (95 wt. %). ^[g]^ Ordinary Portland cement. ^[h]^ Composites comprising elemental sulfur (50 wt. %) and dicyclopentadiene (DCPD) (50 wt. %). ^[i]^ Composites comprising elemental sulfur (50 wt. %), DCPD (25 wt. %), and linseed oil (25 wt. %). ^[j]^ Composites comprising elemental sulfur (50 wt. %), DCPD (25 wt. %), and limonene (25 wt. %). ^[k]^ Not determined.

## Data Availability

Data are contained within the article and Supplementary Materials.

## Supplementary Materials

The following supporting information can be downloaded at https://www.mdpi.com/article/10.3390/molecules29174209/s1. Figure S1. Powder XRD trace of LOS_80_@230 (top) compared to that of alpha sulfur (bottom); Figure S2. Powder XRD trace of LOS_85_@230 (top) compared to that of alpha sulfur (bottom); Figure S3. Powder XRD trace of LOS_90_@230 (top) compared to that of alpha sulfur (bottom); Figure S4. Powder XRD trace of LOS_90_@250 (top) compared to that of alpha sulfur (bottom); Figure S5. Powder XRD trace of LOS_90_@300 (top) compared to that of alpha sulfur (bottom); Figure S6. Portion of the FT-IR spectrum of LOS_80_@230 (grey trace), LOS_85_@230 (orange trace), LOS_90_@230 (blue trace), LOS_90_@250 (yellow trace), and LOS_90_@300 (green trace); Figure S7. Portion of the FT-IR spectrum of LOS_80_@230 (grey trace), LOS_85_@230 (orange trace), LOS_90_@230 (blue trace), LOS_90_@250 (yellow trace), and LOS_90_@300 (green trace); Figure S8. Scanning electron microscopy (SEM) image of LOS_80_@230; Figure S9. Scanning electron microscopy (SEM) image of LOS85@230; Figure S10. Scanning electron microscopy (SEM) image of LOS90@230; Figure S11. Scanning electron microscopy (SEM) image of LOS90@250; Figure S12. Scanning electron microscopy (SEM) image of LOS90@300; Figure S13. Differential scanning calorimetry (DSC) traces (endothermic down) of the first heating (blue line) and first cooling (orange line) cycle for LOS80@230; Figure S14. Differential scanning calorimetry (DSC) traces (endothermic down) of the second heating (blue line) and second cooling (orange line) cycle for LOS80@230; Figure S15. Differential Scanning calorimetry (DSC) traces (endothermic down) of the third heating (blue line) and third cooling (orange line) cycle for LOS80@230; Figure S16. Differential scanning calorimetry (DSC) traces (endothermic down) of the first heating (blue line) and first cooling (orange line) cycle for LOS85@230; Figure S17. Differential scanning calorimetry (DSC) traces (endothermic down) of the second heating (blue line) and second cooling (orange line) cycle for LOS85@230; Figure S18. Differential scanning calorimetry (DSC) traces (endothermic down) of the third heating (blue line) and third cooling (orange line) cycle for LOS85@230; Figure S19. Differential scanning calorimetry (DSC) traces (endothermic down) of the first heating (blue line) and first cooling (orange line) cycle for LOS90@230; Figure S20. Differential scanning calorimetry (DSC) traces (endothermic down) of the second heating (blue line) and second cooling (orange line) cycle for LOS90@230; Figure S21. Differential scanning calorimetry (DSC) traces (endothermic down) of the third heating (blue line) and third cooling (orange line) cycle for LOS90@230; Figure S22. Differential scanning calorimetry (DSC) traces (endothermic down) of the first heating (blue line) and first cooling (orange line) cycle for LOS90@250; Figure S23. Differential scanning calorimetry (DSC) traces (endothermic down) of the second heating (blue line) and second cooling (orange line) cycle for LOS90@250; Figure S24. Differential scanning calorimetry (DSC) traces (endothermic down) of the third heating (blue line) and third cooling (orange line) cycle for LOS90@250; Figure S25. Differential scanning calorimetry (DSC) traces (endothermic down) of the first heating (blue line) and first cooling (orange line) cycle for LOS90@300; Figure S26. Differential scanning calorimetry (DSC) traces (endothermic down) of the second heating (blue line) and second cooling (orange line) cycle for LOS_90_@300; Figure S27. Differential scanning calorimetry (DSC) traces (endothermic down) of the third heating (blue line) and third cooling (orange line) cycle for LOS_90_@300; Figure S28. Representative stress–strain plot for the compressive-strength measurements of LOS_80_@230; Figure S29. Representative stress–strain plot for the compressive-strength measurements of LOS_85_@230; Figure S30. Representative stress–strain plot for the compressive-strength measurements of LOS_90_@230; Figure S31. Representative stress–strain plot for the compressive-strength measurements of LOS_90_@250; Figure S32. Representative stress–strain plot for the compressive-strength measurements of LOS_90_@300; Figure S33. Stress–strain curves of LOS_85_@230 determined during flexural strength testing. The orange line represents the propagations of the linear region of the stress–strain curve; Figure S34. P Stress–strain curves of LOS_90_@230 determined during flexural strength testing. The orange line represents the propagations of the linear region of the stress–strain curve.
