# Supplementary material for: The Influence of the Comonomer Ratio and Reaction Temperature on the Mechanical, Thermal, and Morphological Properties of Lignin Oil–Sulfur Composites
Source: Molecules. 2024 Sep 5;29(17):4209. doi: 10.3390/molecules29174209 (PMC11397338; doi:10.3390/molecules29174209)
Supplement: Supplementary file 1 [file molecules-29-04209-s001.zip › molecules-3094933-supplementary.pdf]

Supplementary Materials

The Influence of the Comonomer Ratio and Reaction Temperature on the Mechanical, Thermal, and Morphological Properties of Lignin Oil–Sulfur Composites

Katelyn A. Tisdale, Nawoda L. Kapuge Dona, and Rhett C. Smith *

Department of Chemistry and Center for Optical Materials Science and Engineering Technology, Clemson University, Clemson, SC 29634, USA

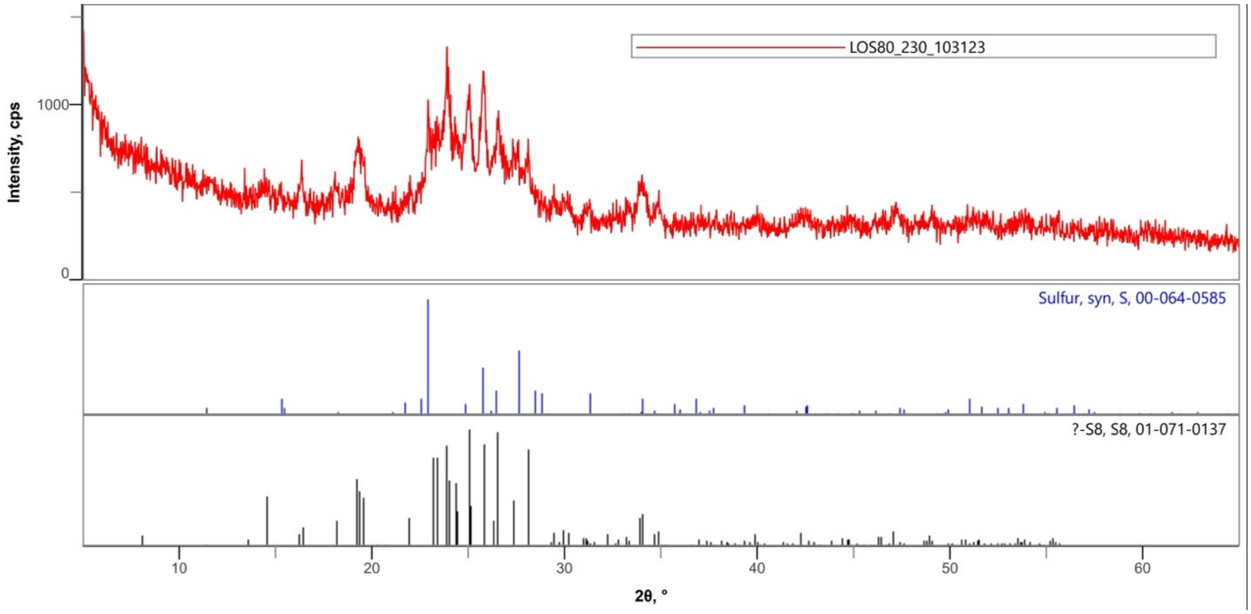

Figure S1. Powder XRD trace of LOS_{80}@230 (top) compared to that of alpha sulfur (bottom).

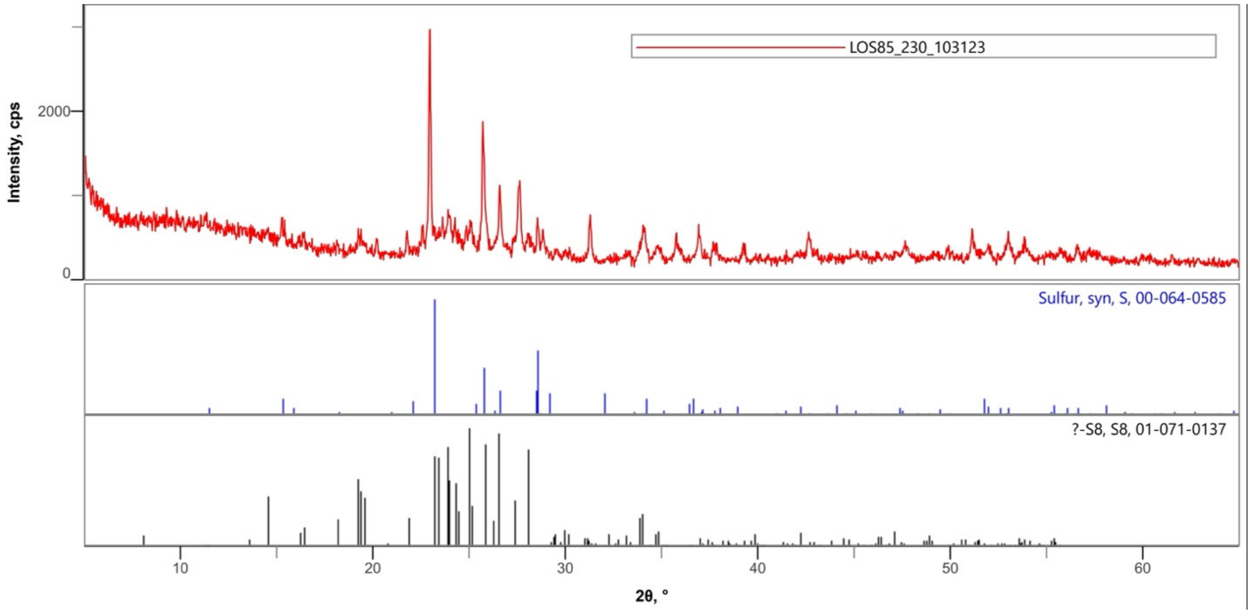

Figure S2. Powder XRD trace of LOS_{85}@230 (top) compared to that of alpha sulfur (bottom).

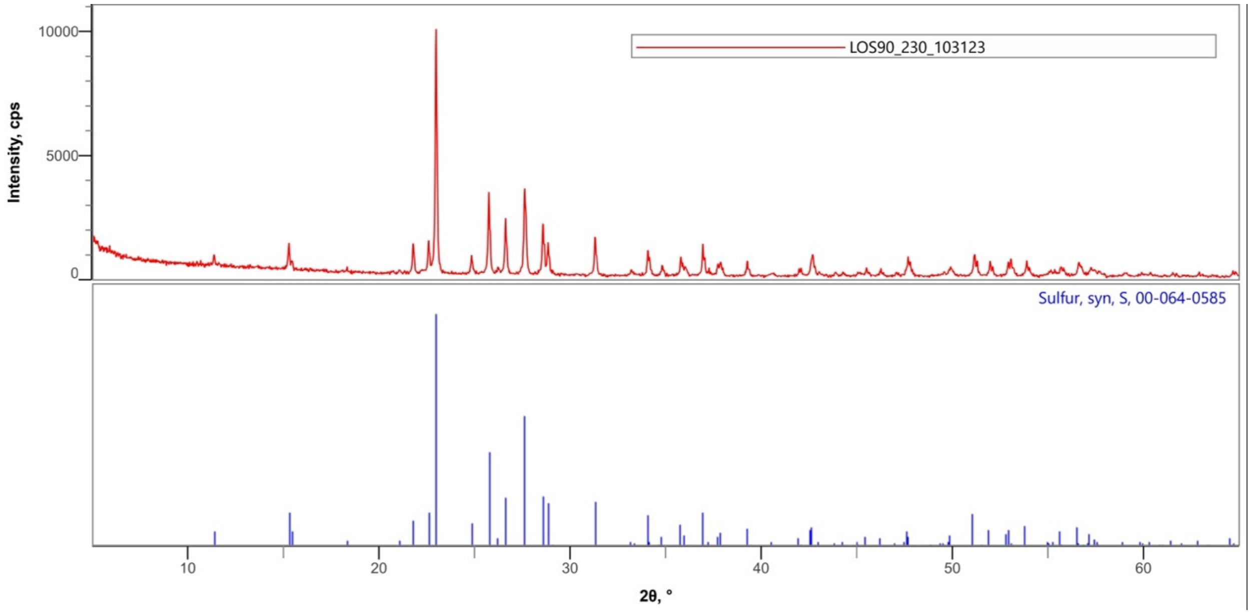

Figure S3. Powder XRD trace of LOS_{90}@230 (top) compared to that of alpha sulfur (bottom).

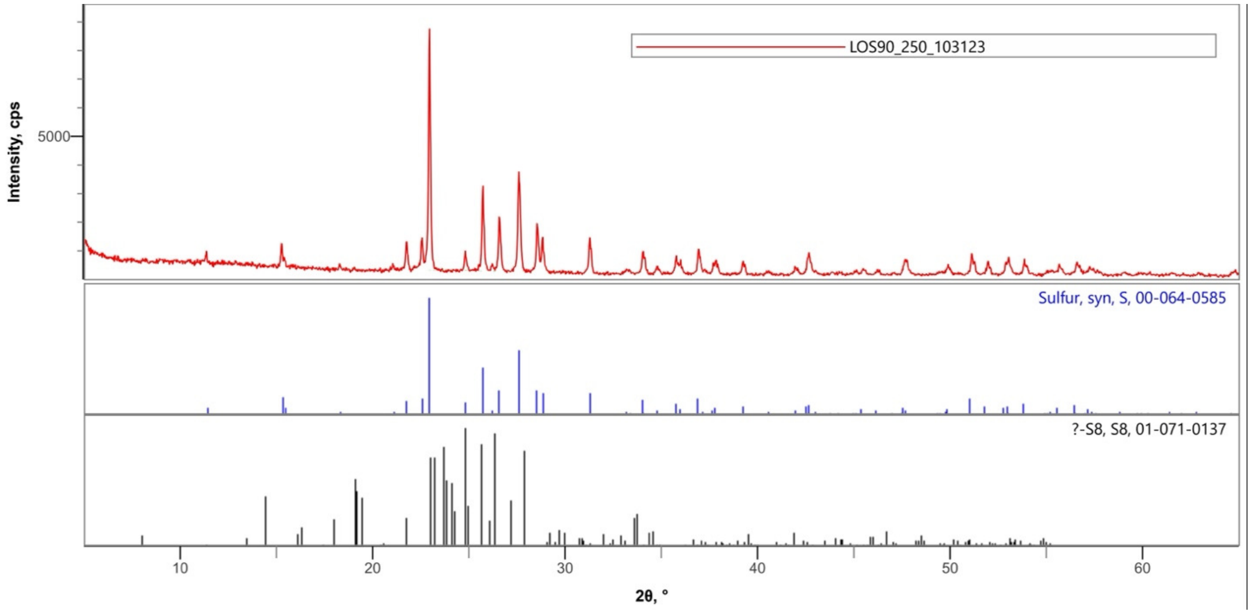

Figure S4. Powder XRD trace of LOS_{90}@250 (top) compared to that of alpha sulfur (bottom).

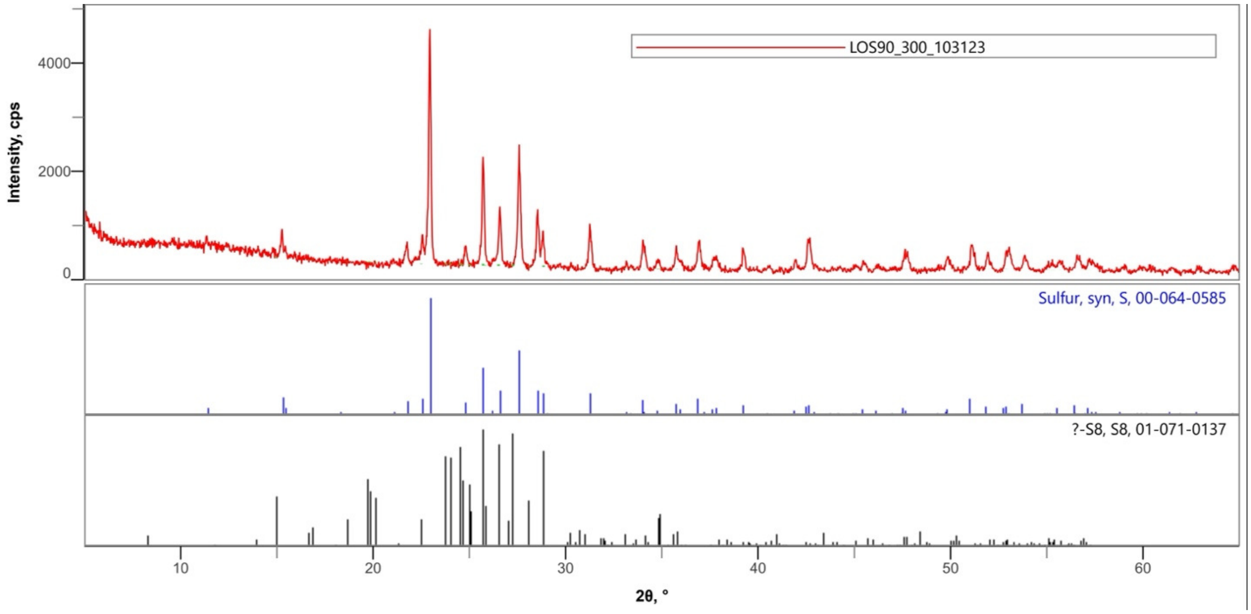

Figure S5. Powder XRD trace of LOS_{90}@300 (top) compared to that of alpha sulfur (bottom).

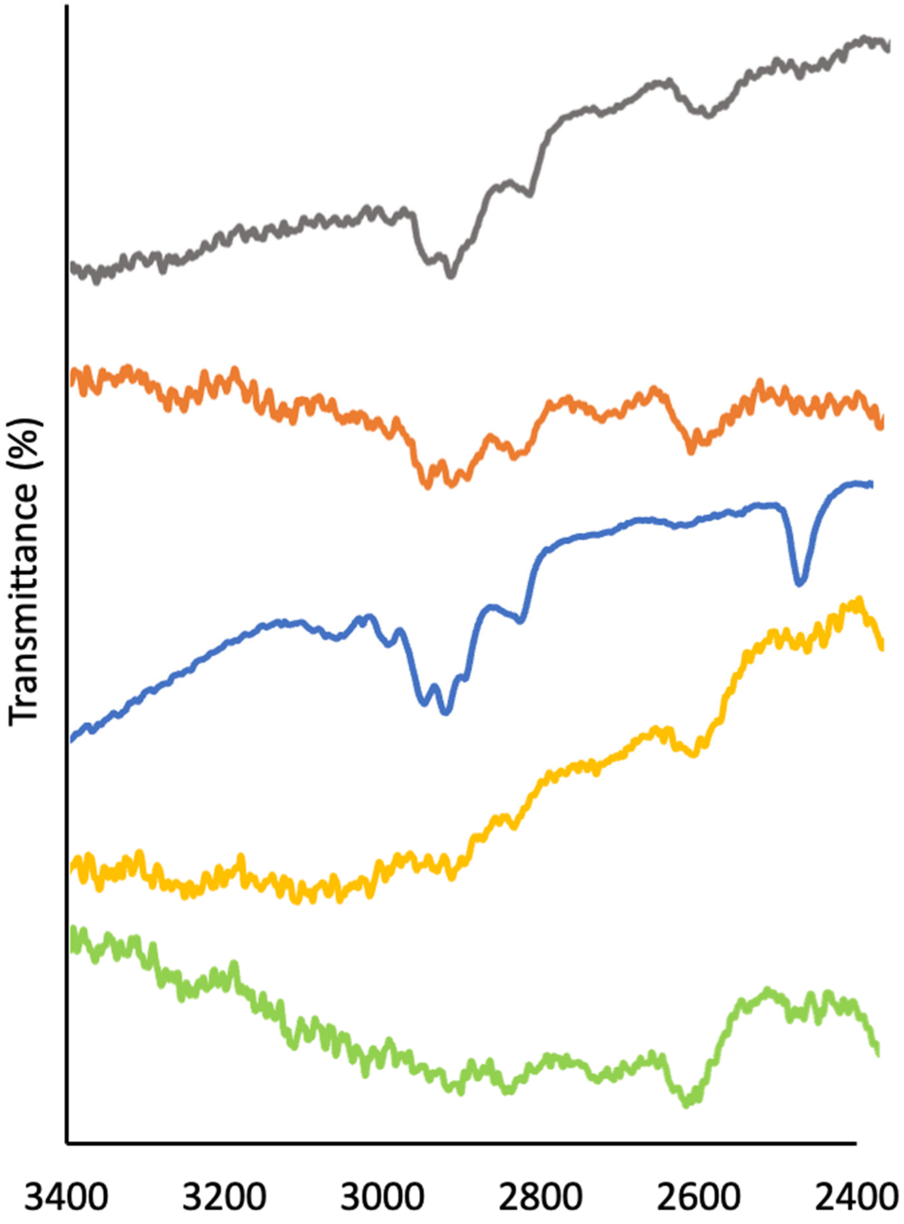

Figure S6. Portion of FT-IR spectrum of LOS_{80}@230 (grey trace), LOS_{85}@230 (orange trace), LOS_{90}@230 (blue trace), LOS_{90}@250 (yellow trace), and LOS_{90}@300 (green trace).

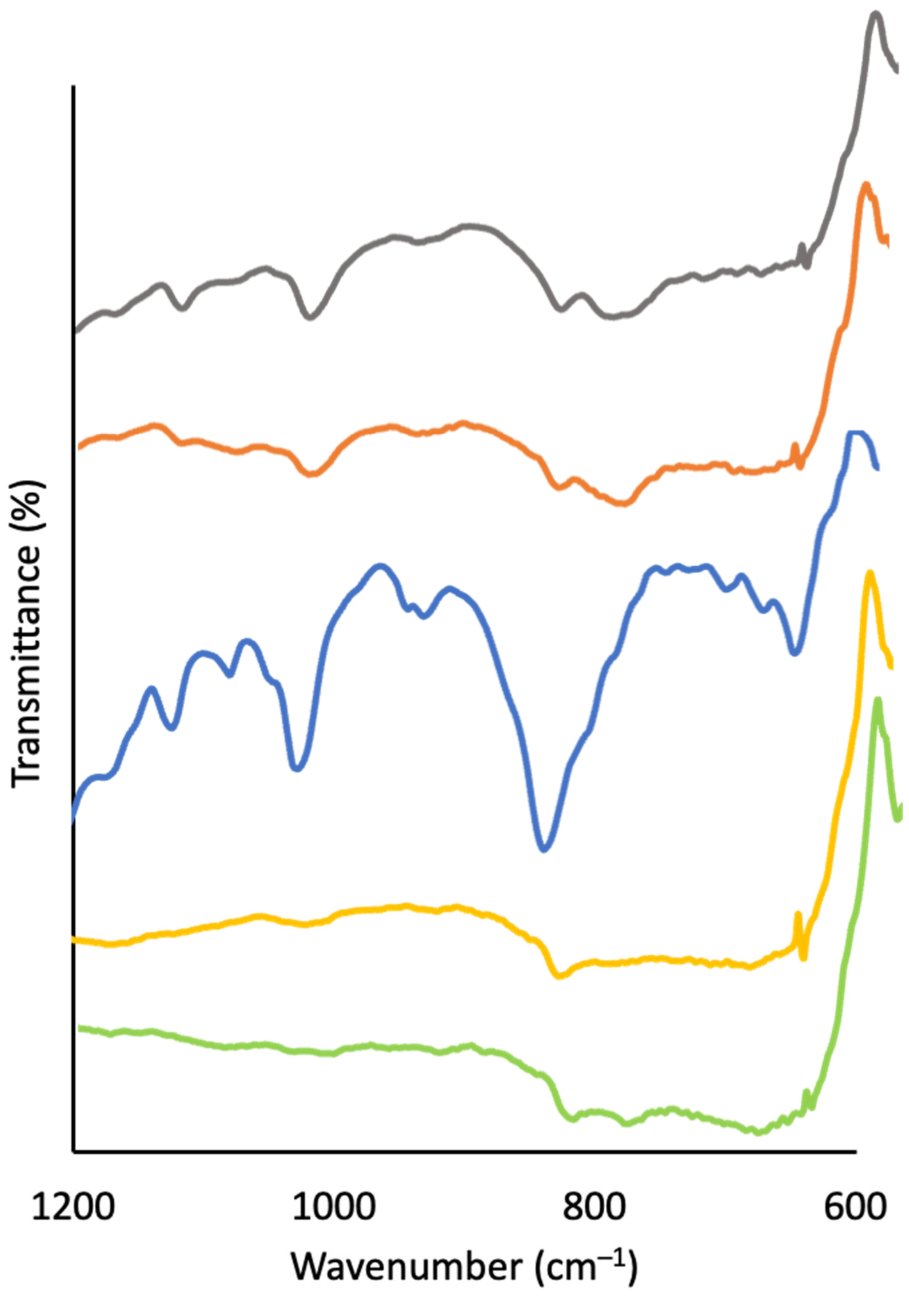

Figure S7. Portion of FT-IR spectrum of LOS_{80}@230 (grey trace), LOS_{85}@230 (orange trace), LOS_{90}@230 (blue trace), LOS_{90}@250 (yellow trace), and LOS_{90}@300 (green trace).

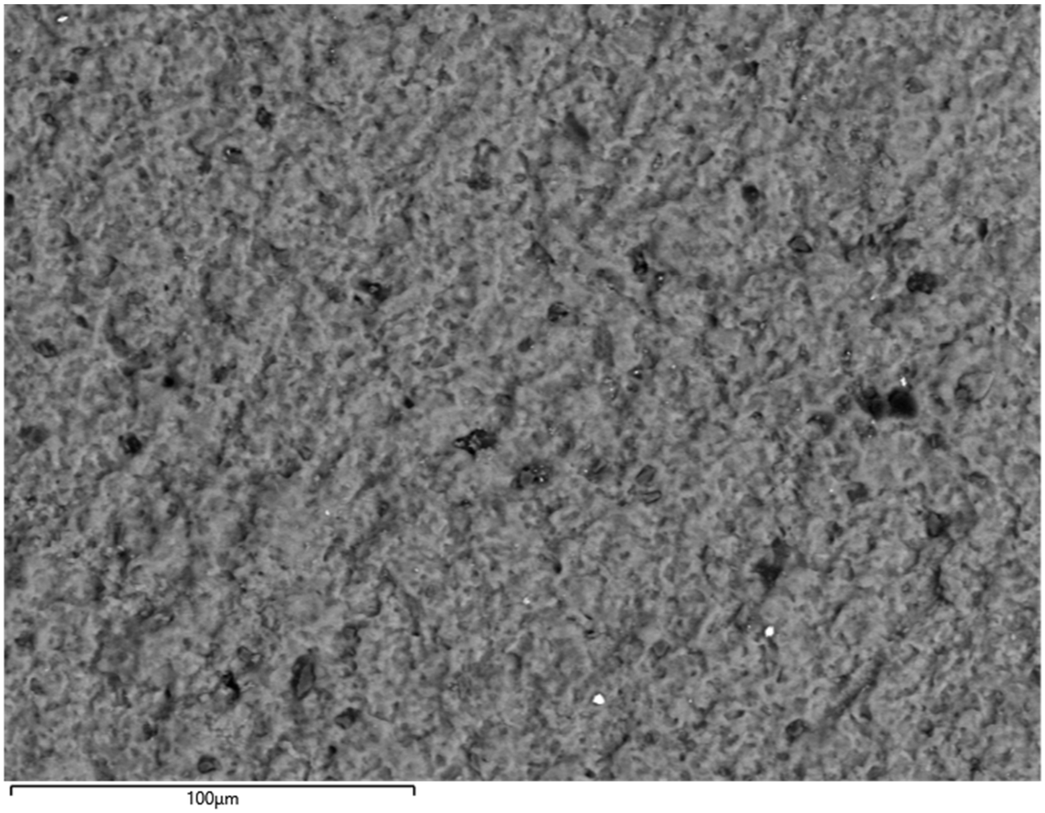

Figure S8. Scanning electron microscopy (SEM) image of LOS_{80}@230.

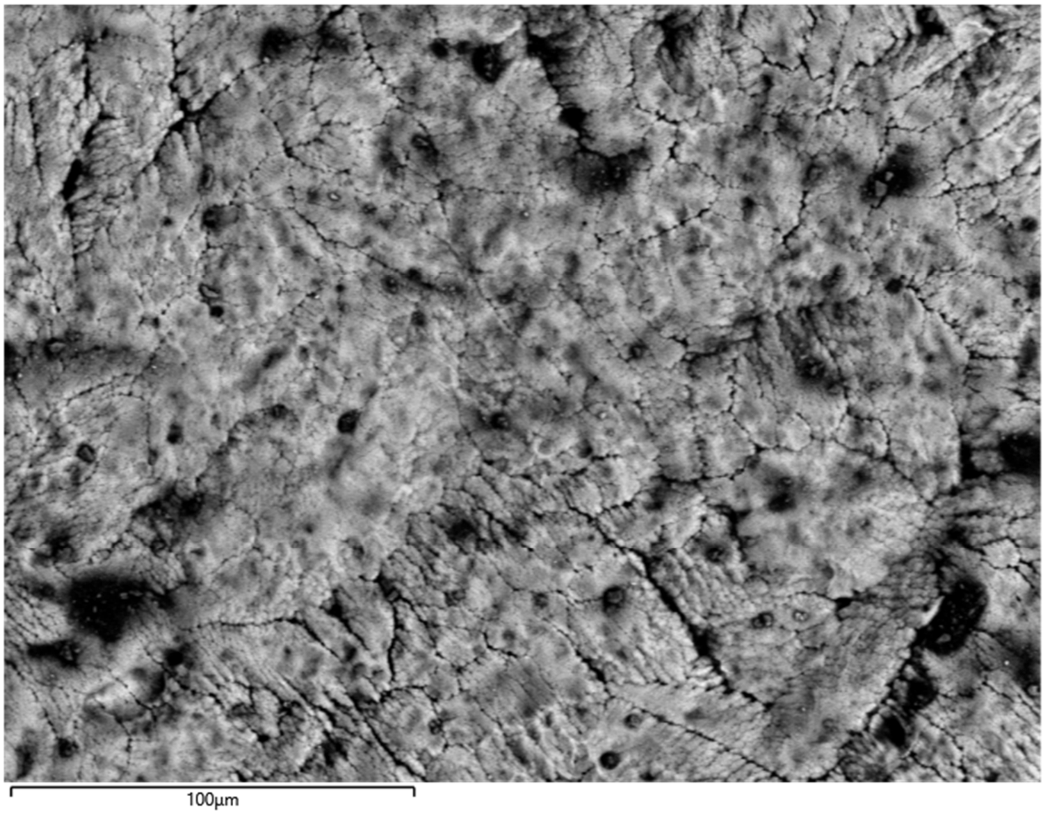

Figure S9. Scanning electron microscopy (SEM) image of LOS_{85}@230.

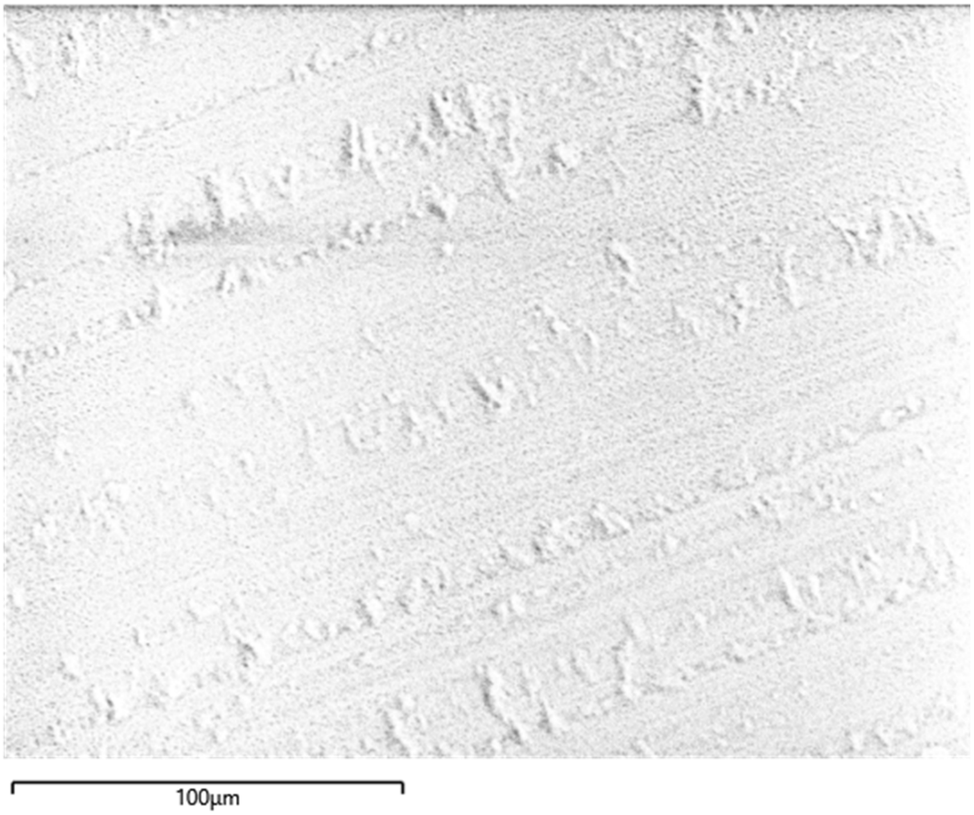

Figure S10. Scanning electron microscopy (SEM) image of LOS_{90}@230.

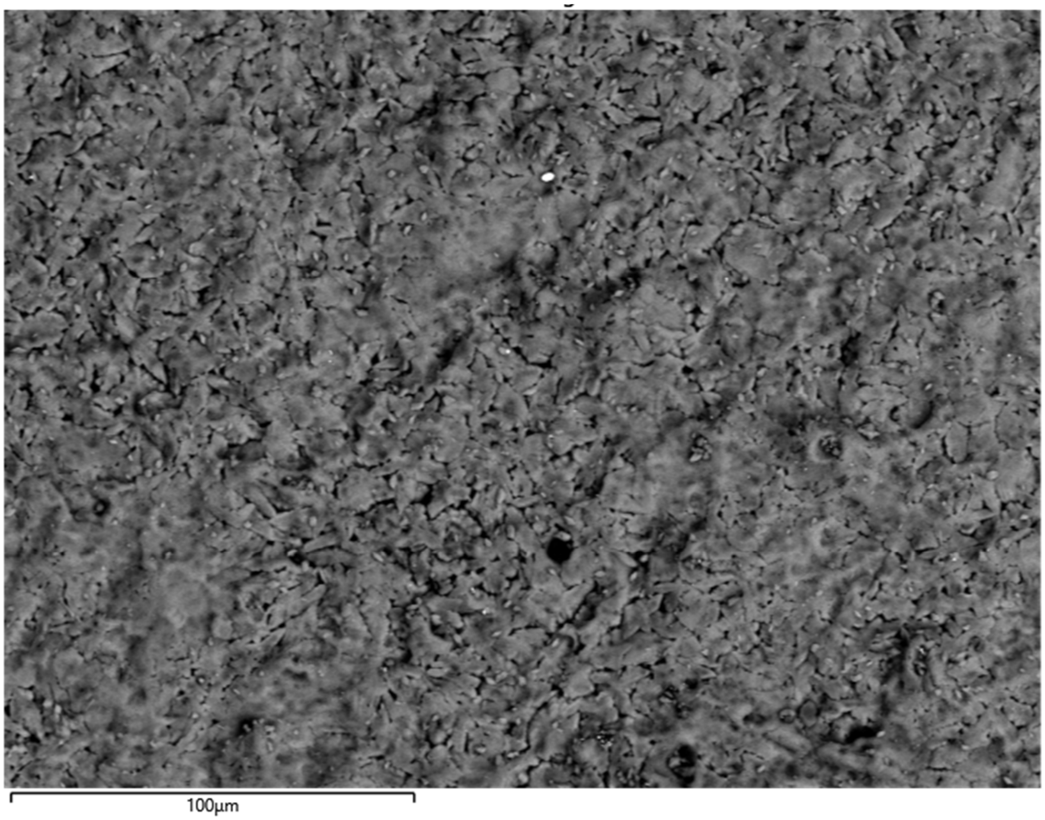

Figure S11. Scanning electron microscopy (SEM) image of LOS_{90}@250.

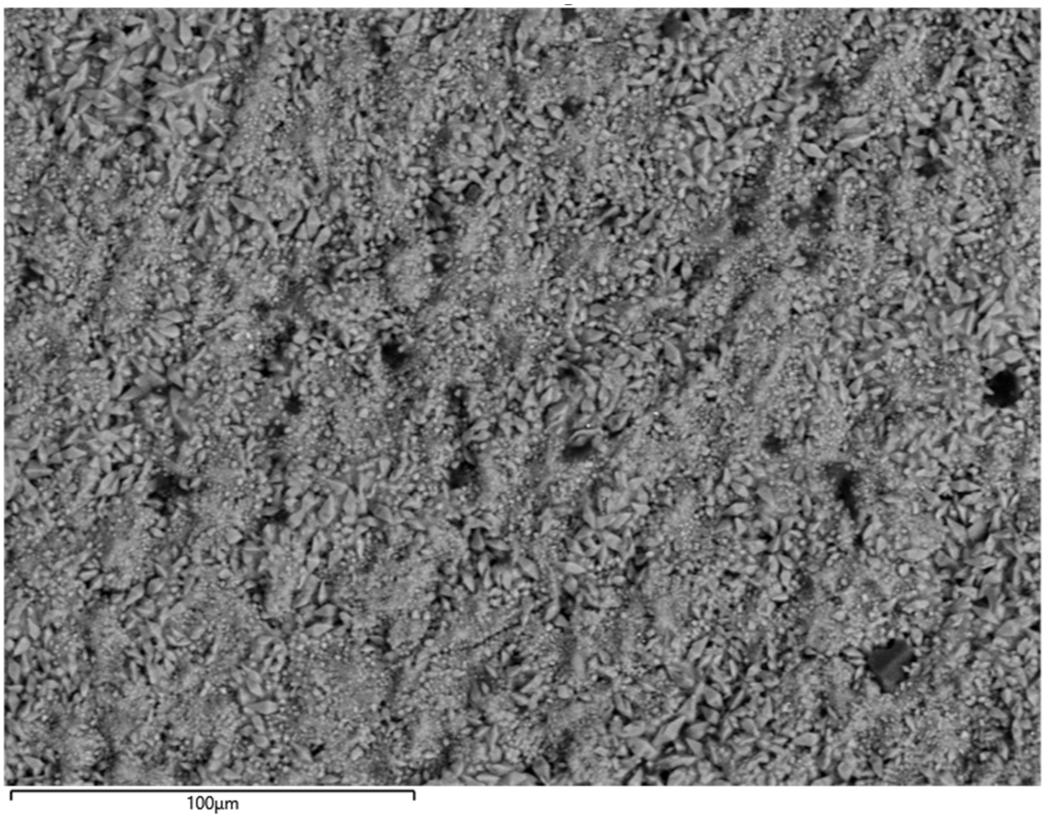

Figure S12. Scanning electron microscopy (SEM) image of LOS_{90}@300.

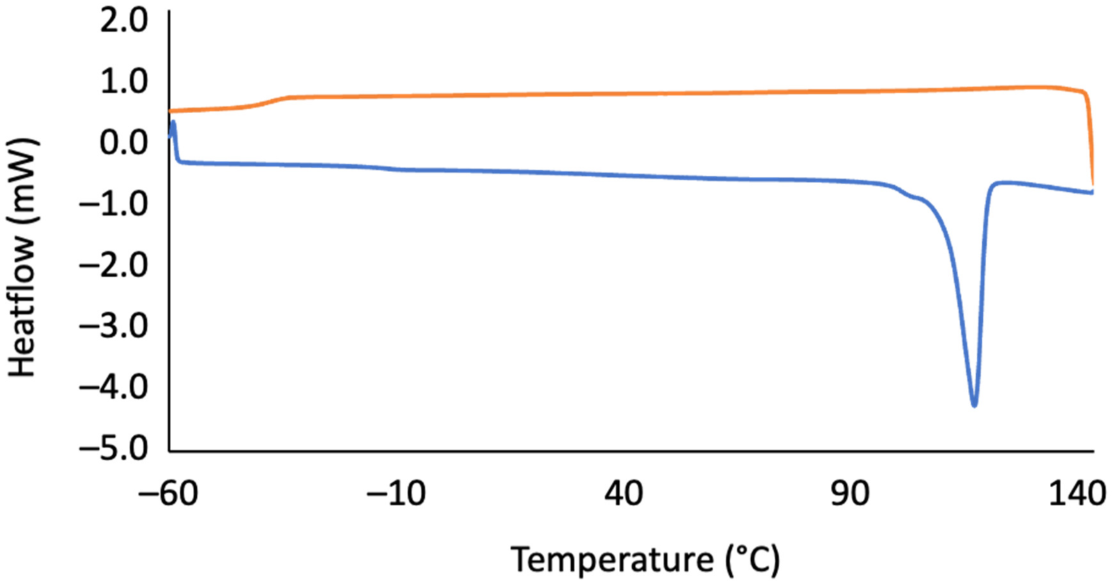

Figure S13. Differential Scanning calorimetry (DSC) traces (endothermic down) of the first heating (blue line) and first cooling (orange line) cycle for LOS_{80}@230.

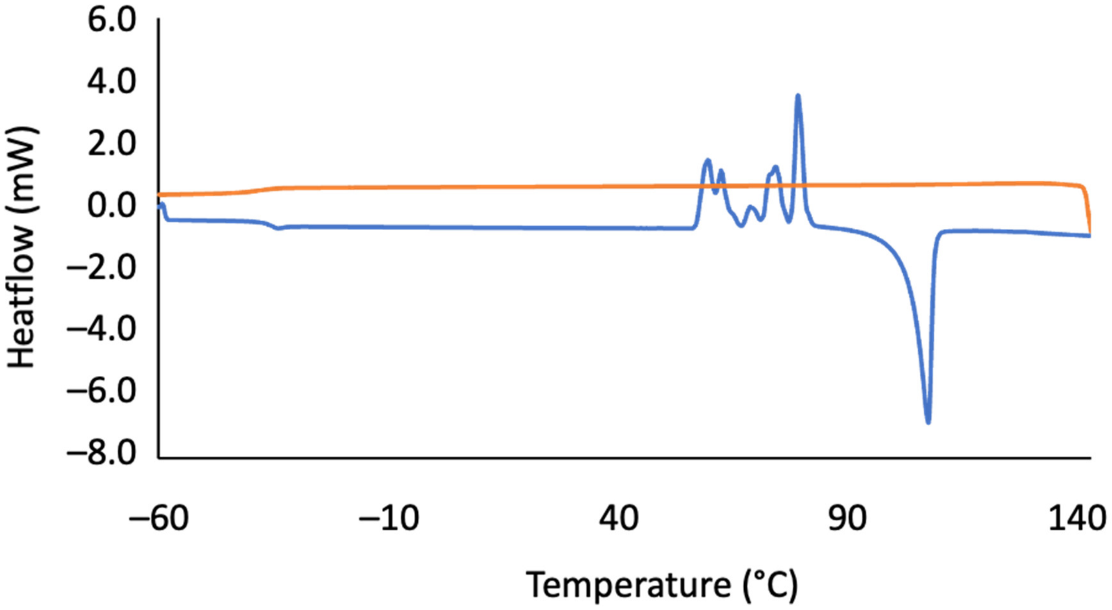

Figure S14. Differential Scanning calorimetry (DSC) traces (endothermic down) of the second heating (blue line) and second cooling (orange line) cycle for LOS_{80}@230.

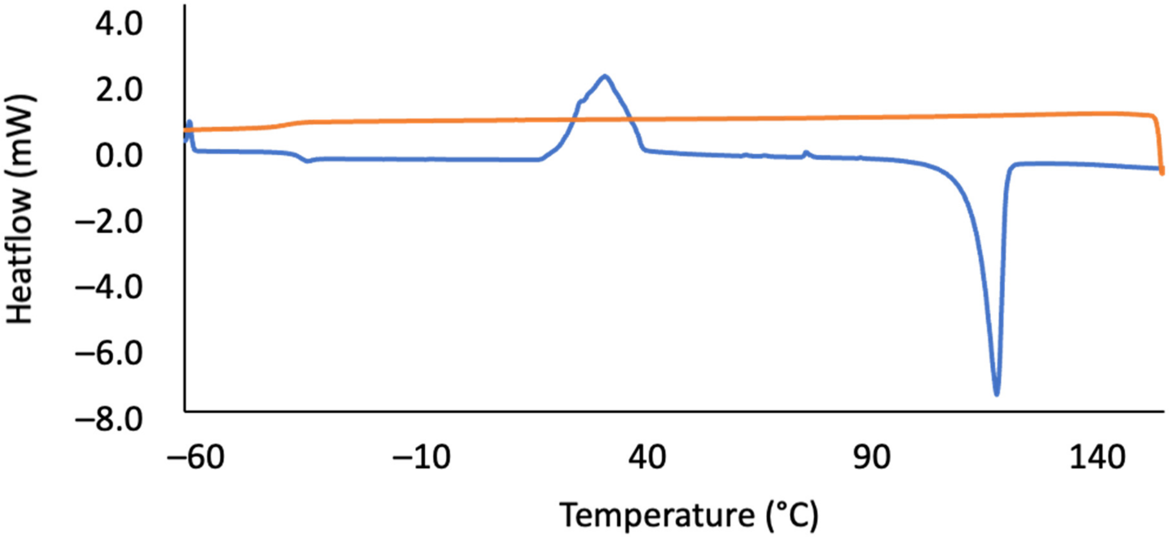

Figure S15. Differential Scanning calorimetry (DSC) traces (endothermic down) of the third heating (blue line) and third cooling (orange line) cycle for LOS_{80}@230.

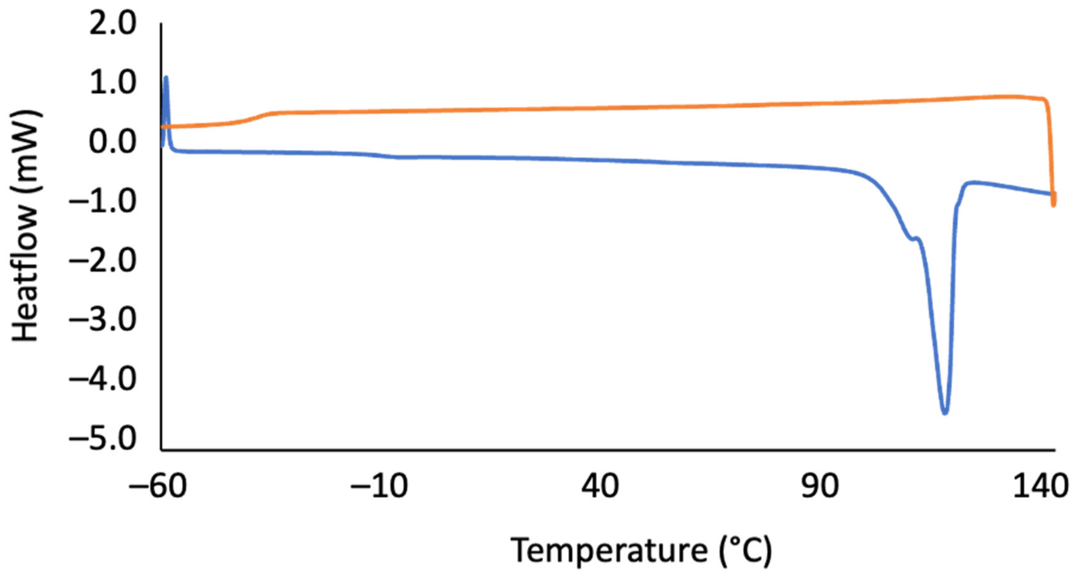

Figure S16. Differential Scanning calorimetry (DSC) traces (endothermic down) of the first heating (blue line) and first cooling (orange line) cycle for LOS_{85}@230.

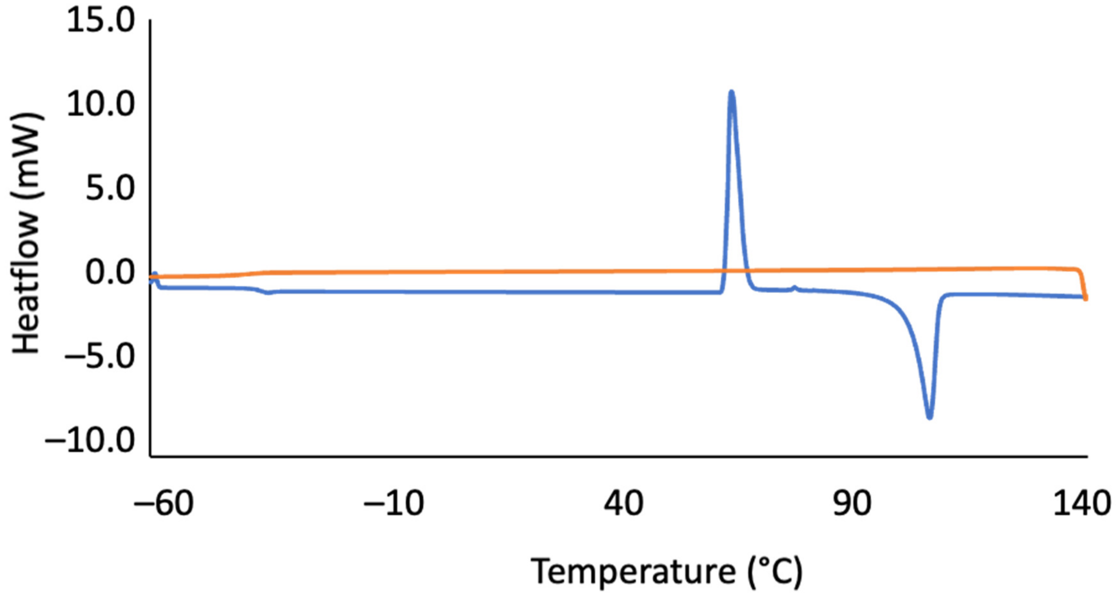

Figure S17. Differential Scanning calorimetry (DSC) traces (endothermic down) of the second heating (blue line) and second cooling (orange line) cycle for LOS_{85}@230.

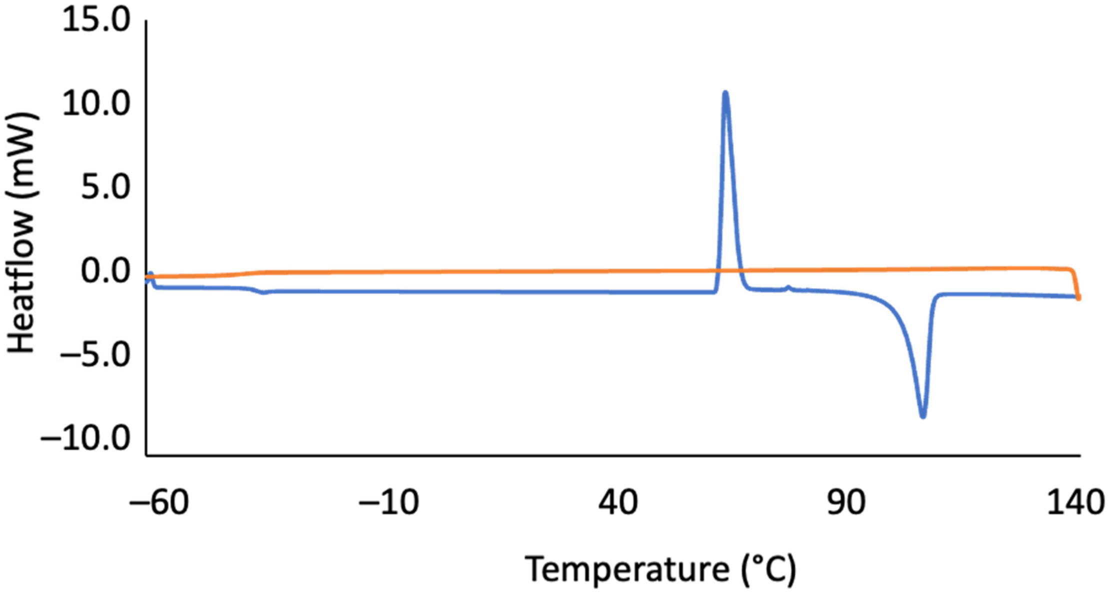

Figure S18. Differential Scanning calorimetry (DSC) traces (endothermic down) of the third heating (blue line) and third cooling (orange line) cycle for LOS_{85}@230.

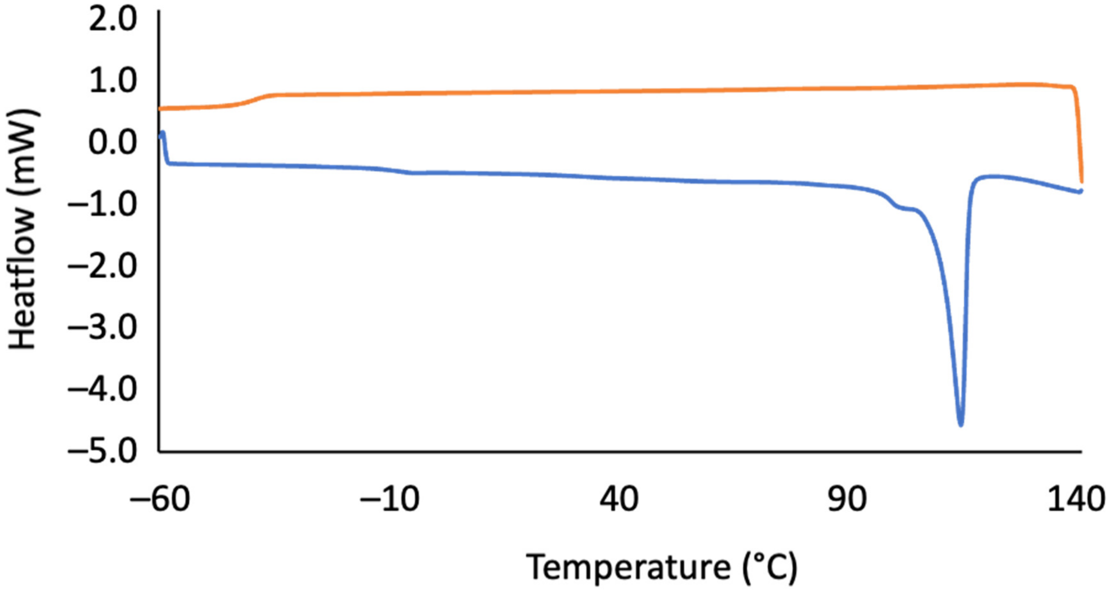

Figure S19. Differential Scanning calorimetry (DSC) traces (endothermic down) of the first heating (blue line) and first cooling (orange line) cycle for LOS_{90}@230.

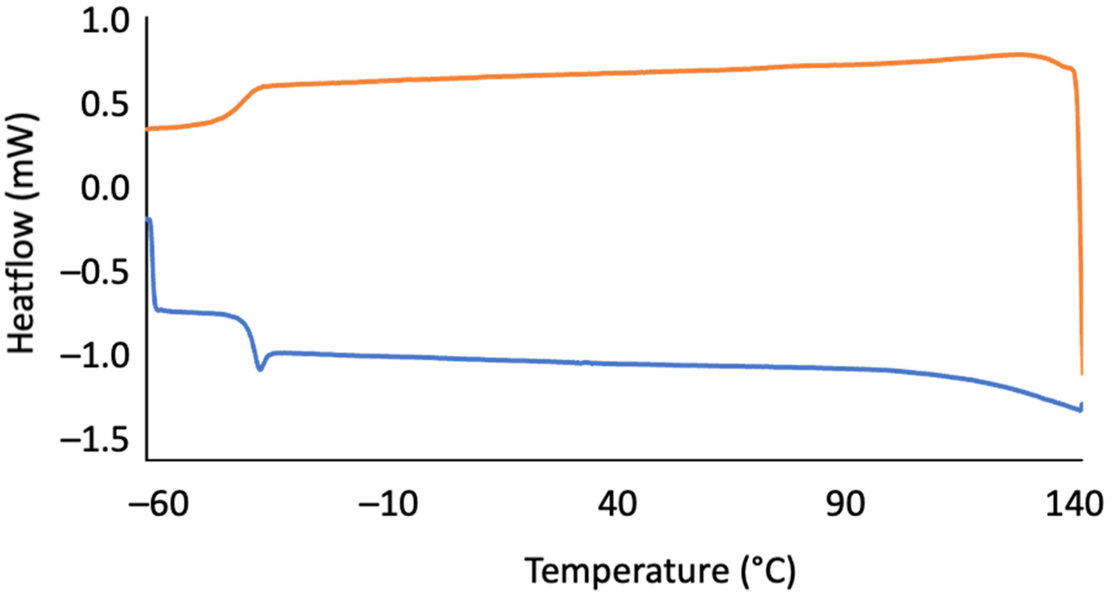

Figure S20. Differential Scanning calorimetry (DSC) traces (endothermic down) of the second heating (blue line) and second cooling (orange line) cycle for LOS_{90}@230.

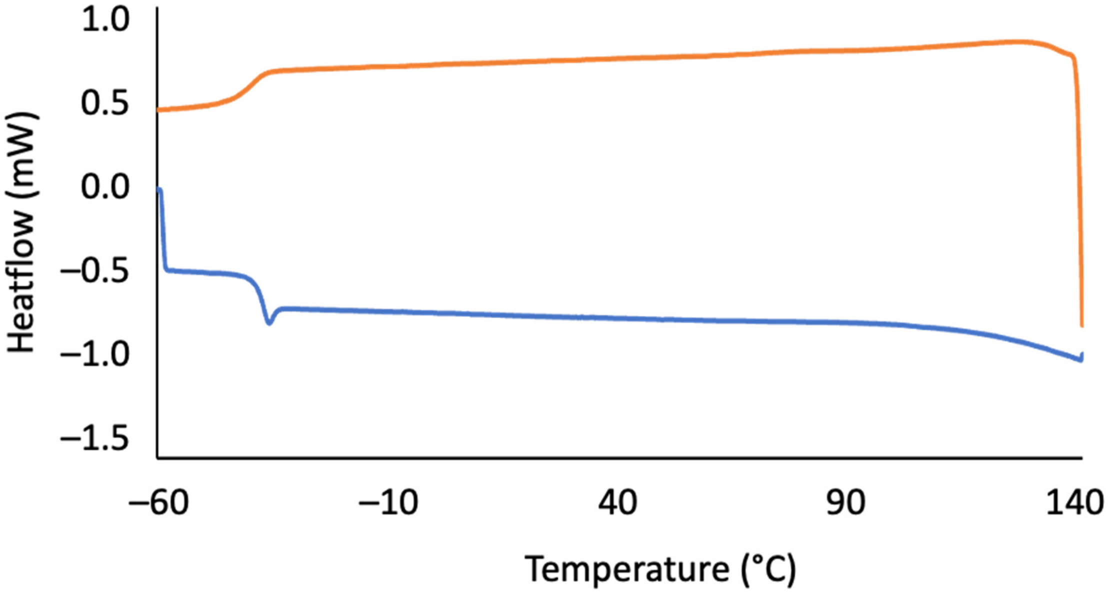

Figure S21. Differential Scanning calorimetry (DSC) traces (endothermic down) of the third heating (blue line) and third cooling (orange line) cycle for LOS_{90}@230.

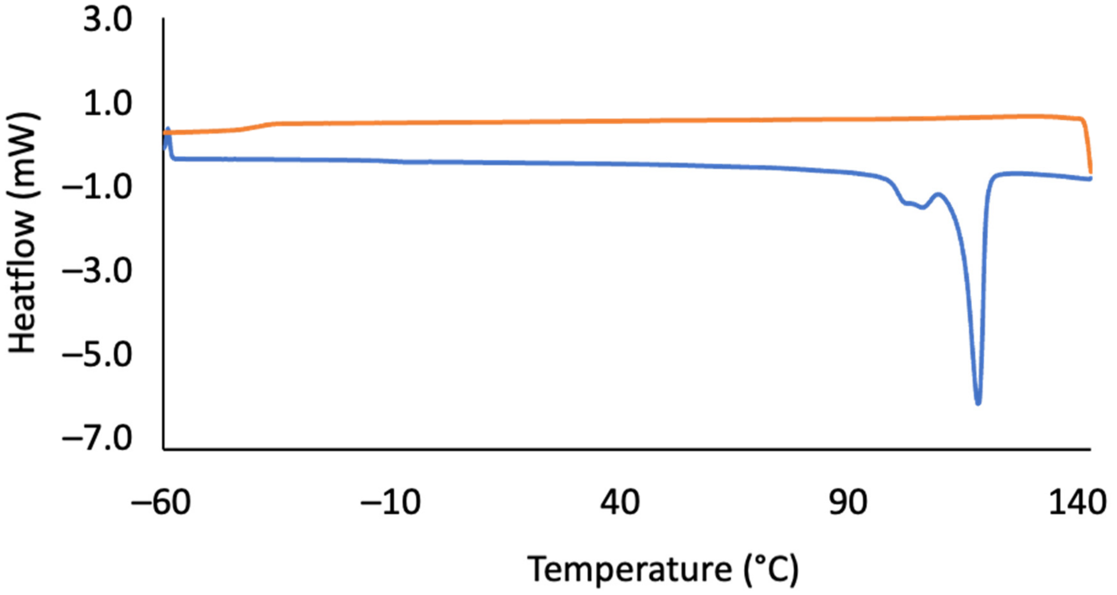

Figure S22. Differential Scanning calorimetry (DSC) traces (endothermic down) of the first heating (blue line) and first cooling (orange line) cycle for LOS_{90}@250.

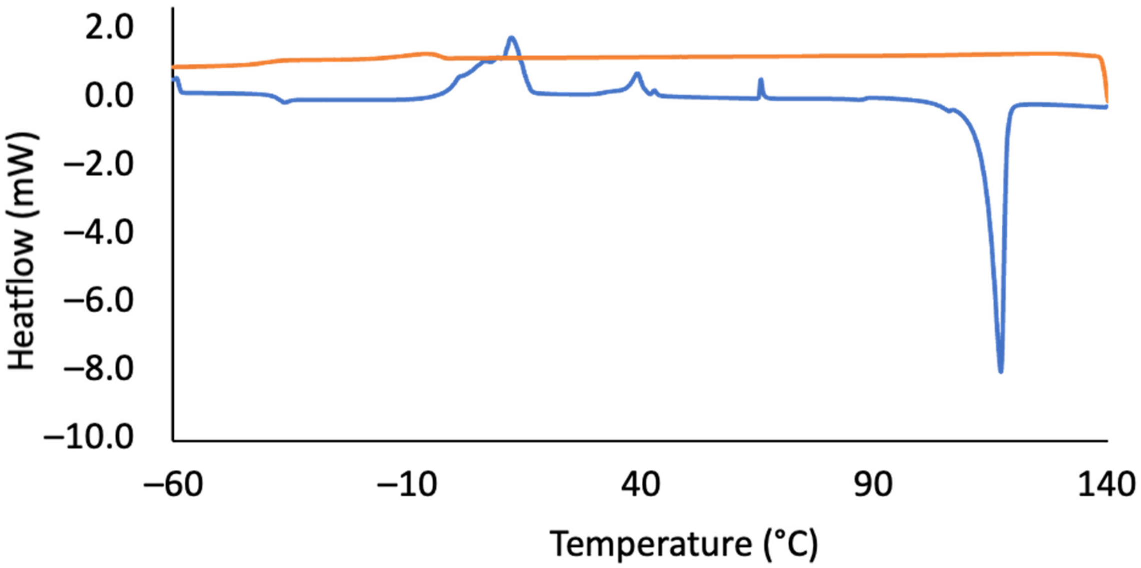

Figure S23. Differential Scanning calorimetry (DSC) traces (endothermic down) of the second heating (blue line) and second cooling (orange line) cycle for LOS_{90}@250.

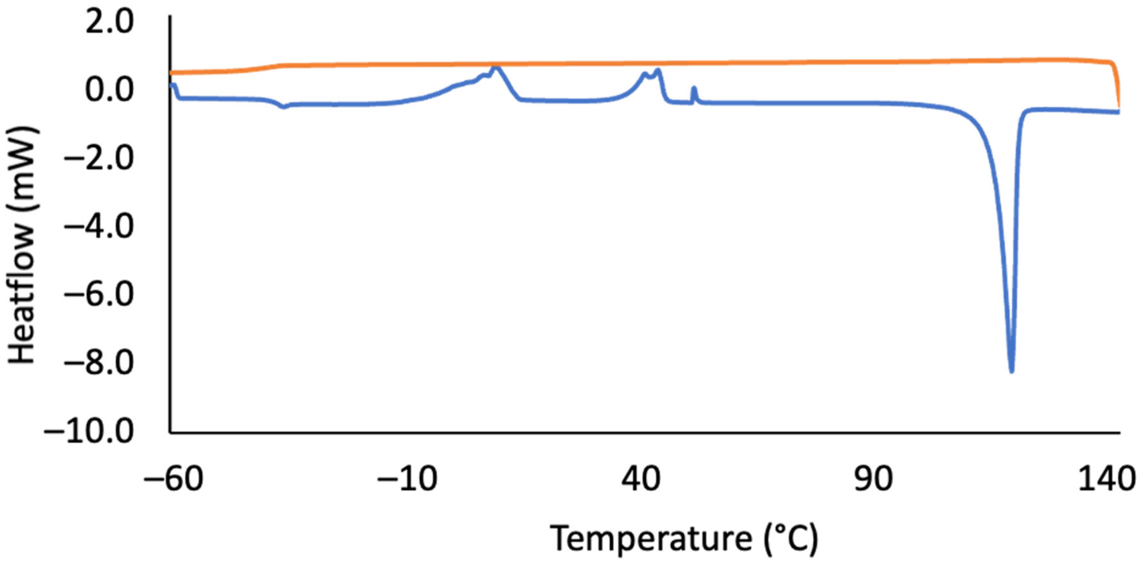

Figure S24. Differential Scanning calorimetry (DSC) traces (endothermic down) of the third heating (blue line) and third cooling (orange line) cycle for LOS_{90}@250.

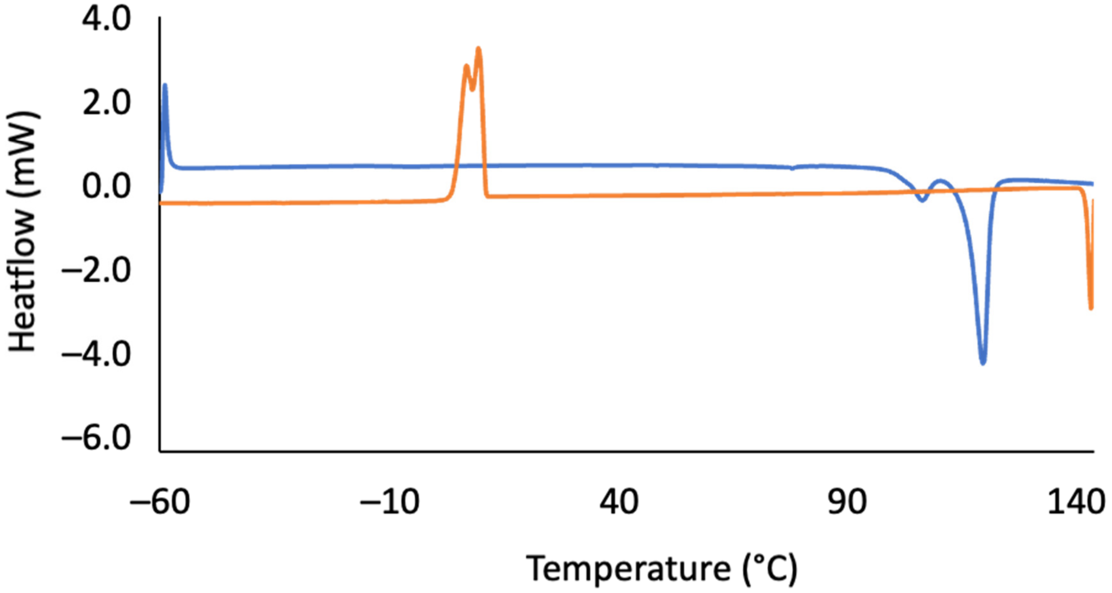

Figure S25. Differential Scanning calorimetry (DSC) traces (endothermic down) of the first heating (blue line) and first cooling (orange line) cycle for LOS_{90}@300.

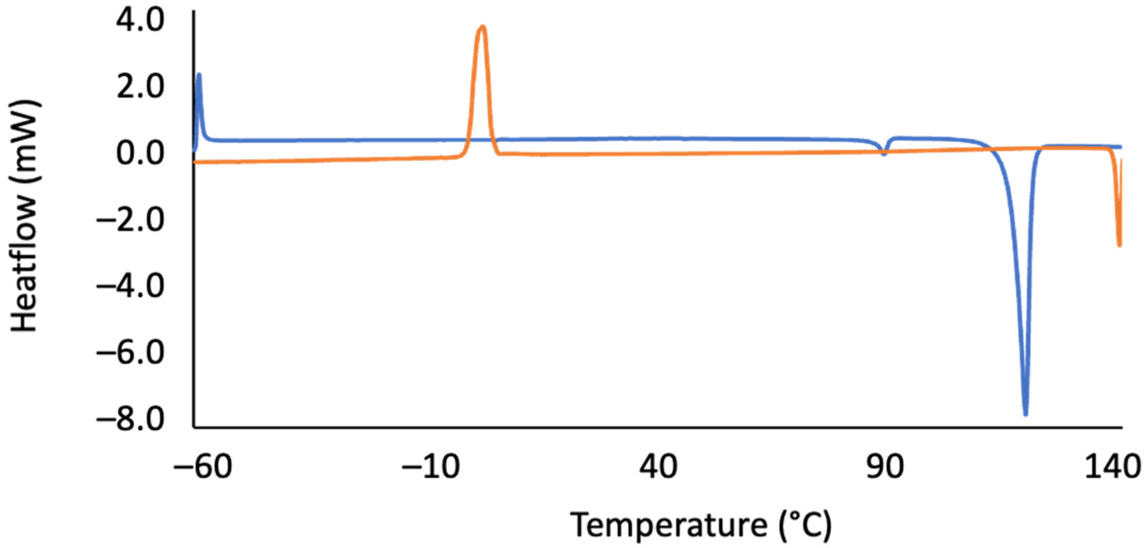

Figure S26. Differential Scanning calorimetry (DSC) traces (endothermic down) of the second heating (blue line) and second cooling (orange line) cycle for LOS_{90}@300.

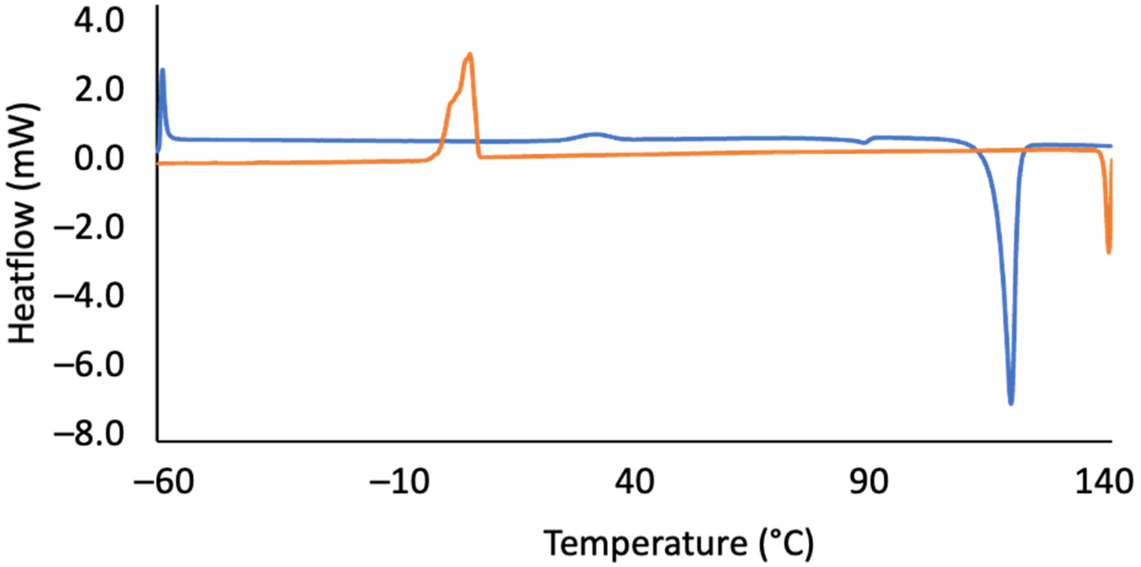

Figure S27. Differential Scanning calorimetry (DSC) traces (endothermic down) of the third heating (blue line) and third cooling (orange line) cycle for LOS_{90}@300.

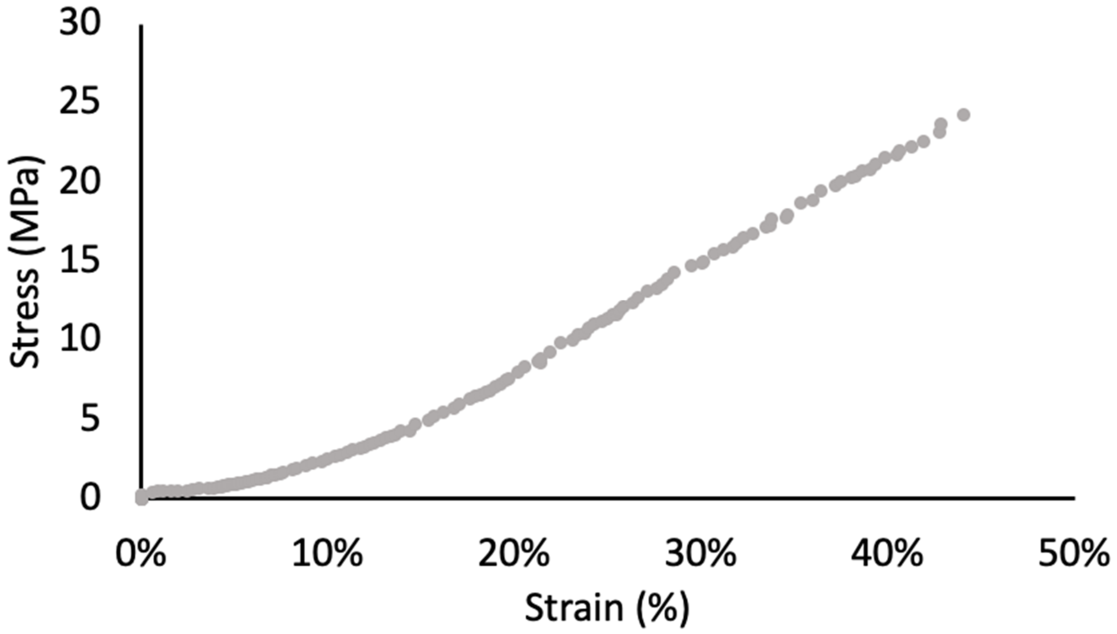

Figure S28. Representative stress-strain plot for the compressive strength measurements of LOS_{80}@230.

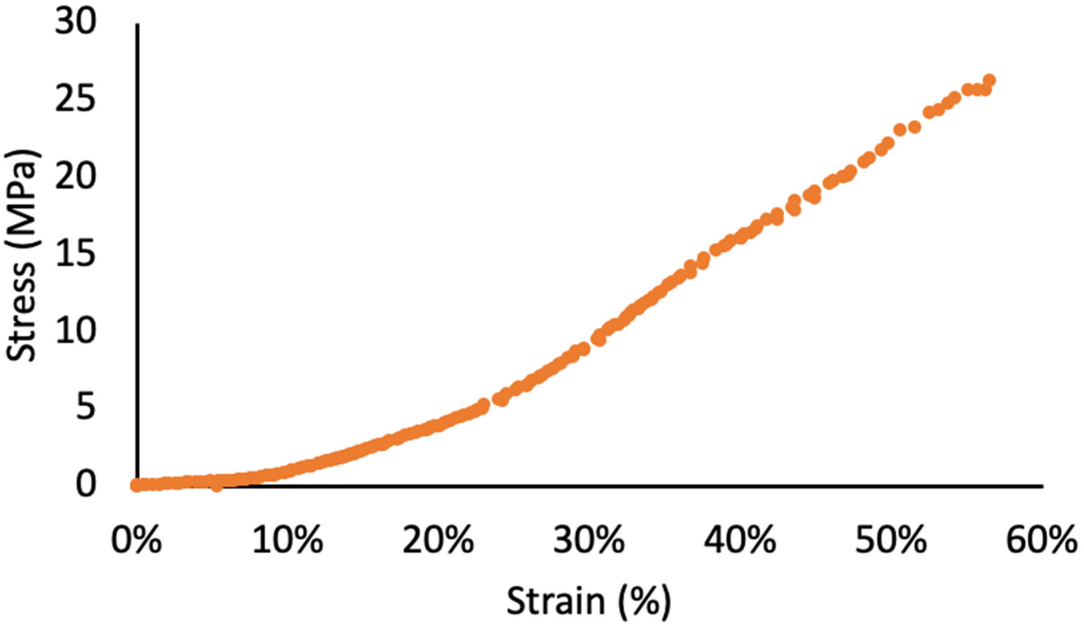

Figure S29. Representative stress-strain plot for the compressive strength measurements of LOS_{85}@230.

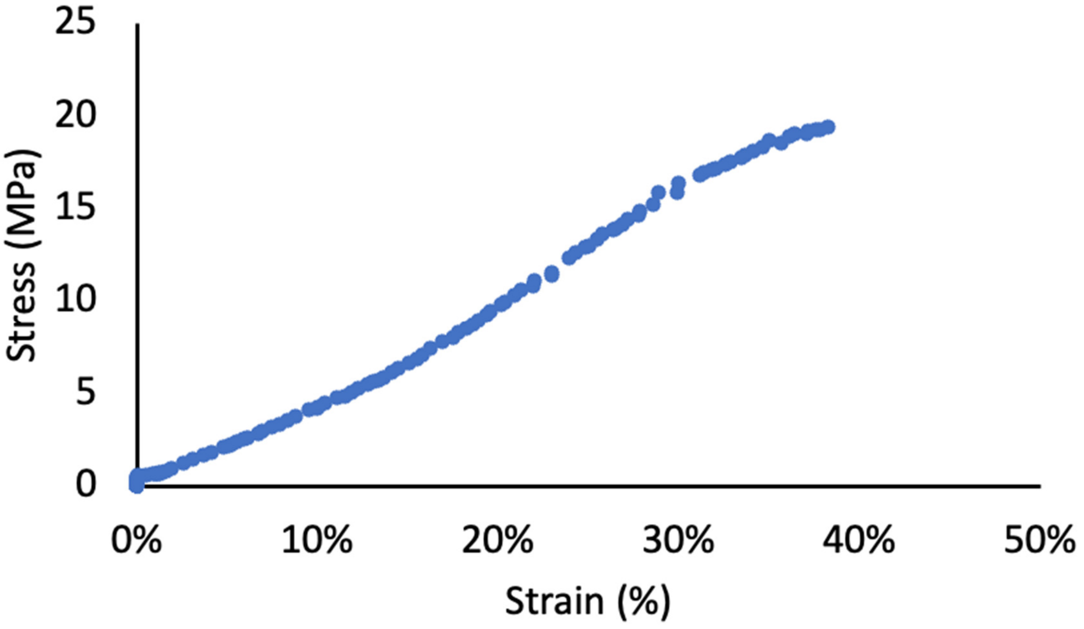

Figure S30. Representative stress-strain plot for the compressive strength measurements of LOS_{90}@230.

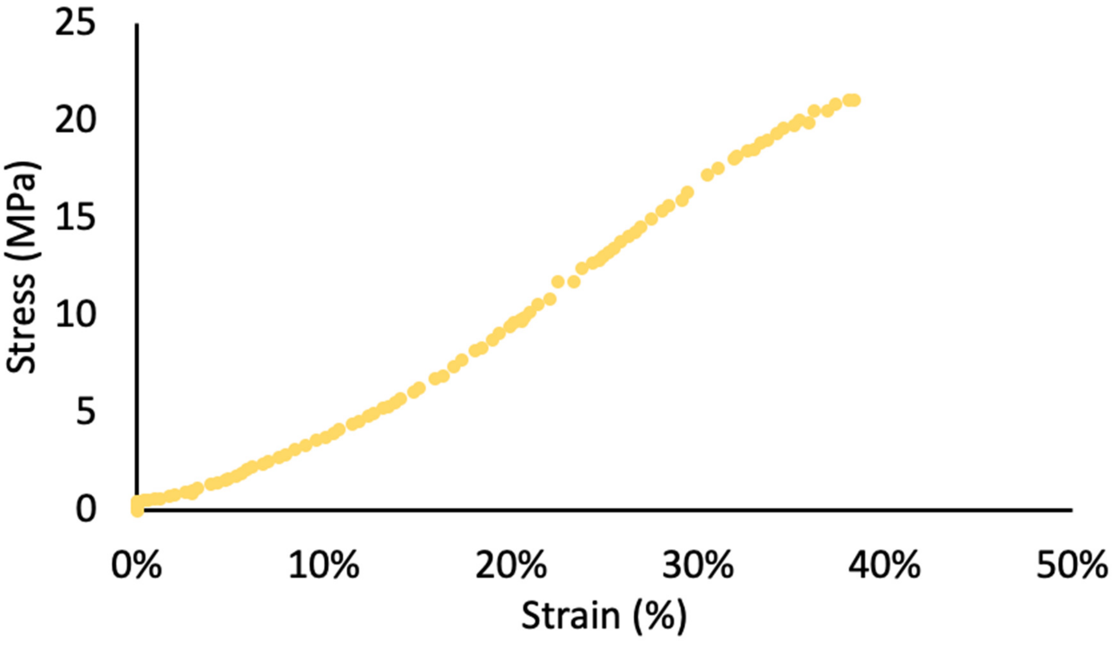

Figure S31. Representative stress-strain plot for the compressive strength measurements of LOS_{90}@250.

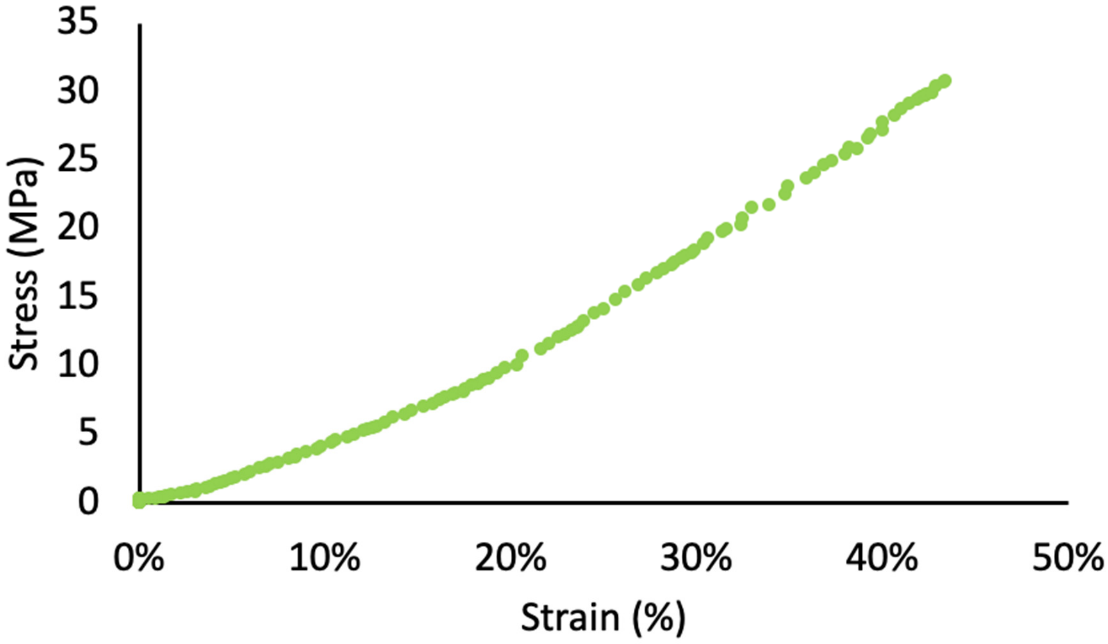

Figure S32. Representative stress-strain plot for the compressive strength measurements of LOS_{90}@300.

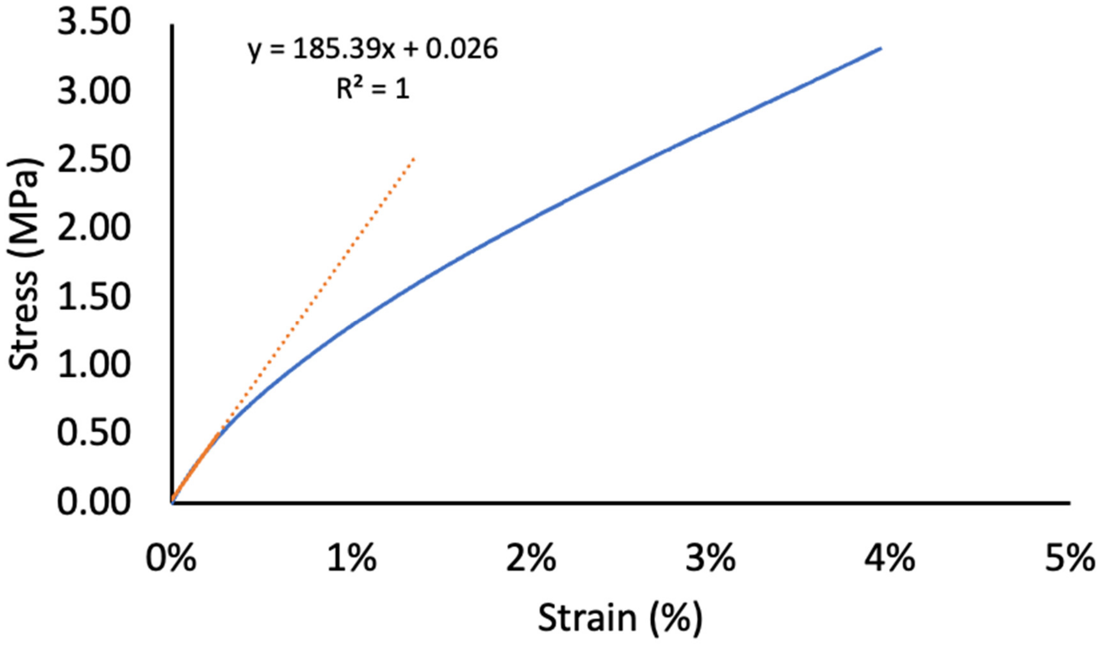

Figure S33. Stress-strain curves of LOS_{85}@230 determined during flexural strength testing. The orange line represents the propagations of the linear region of the stress-strain curve.

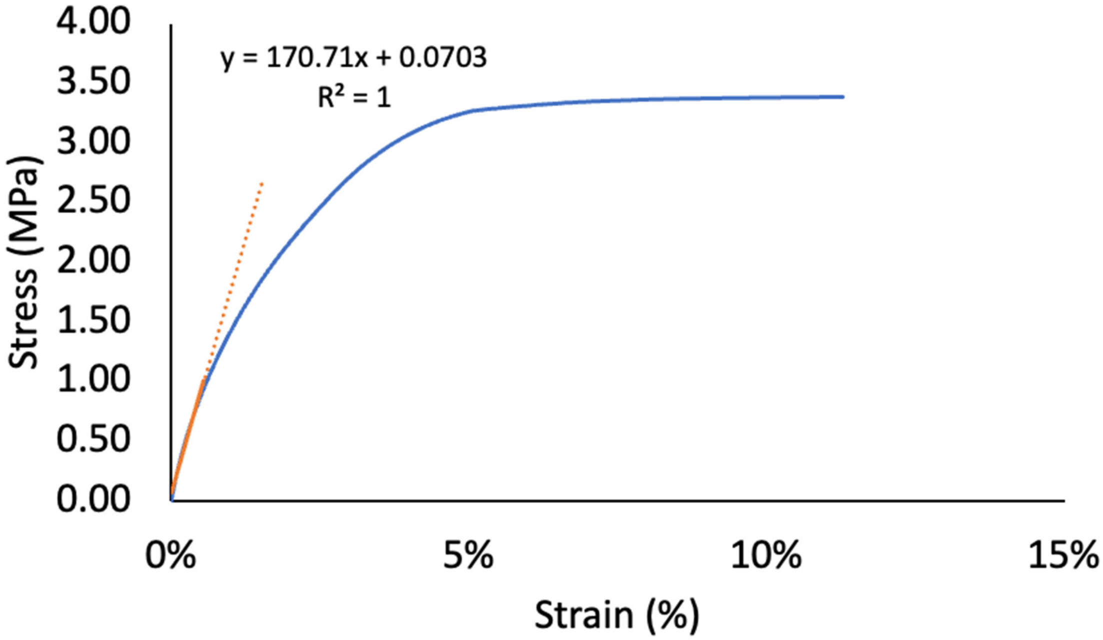

Figure S34. Stress-strain curves of LOS_{90}@230 determined during flexural strength testing. The orange line represents the propagations of the linear region of the stress-strain curve.
